# Cortical and trabecular mechanical properties in the femoral neck vary differently with changes in bone mineral density

**DOI:** 10.1093/jbmrpl/ziae049

**Published:** 2024-04-09

**Authors:** Martin Bittner-Frank, Andreas G Reisinger, Orestis G Andriotis, Dieter H Pahr, Philipp J Thurner

**Affiliations:** Division of Biomechanics, Karl Landsteiner University of Health Sciences, A-3500 Krems an der Donau, Austria; Division of Biomechanics, Karl Landsteiner University of Health Sciences, A-3500 Krems an der Donau, Austria; Institute of Lightweight Design and Structural Biomechanics, TU Wien, A-1060 Vienna, Austria; Division of Biomechanics, Karl Landsteiner University of Health Sciences, A-3500 Krems an der Donau, Austria; Institute of Lightweight Design and Structural Biomechanics, TU Wien, A-1060 Vienna, Austria; Institute of Lightweight Design and Structural Biomechanics, TU Wien, A-1060 Vienna, Austria

**Keywords:** biomechanics, bone matrix, bone μCT, fracture risk assessment, osteoporosis

## Abstract

Osteoporosis is an increasing burden for our aging society. Fracture risk assessment tool (FRAX) and areal bone mineral density (aBMD) have been mainly used as a surrogate, but only identify 46% of patients sustaining a hip fracture. Adding information about material and mechanical properties might improve the fracture risk prediction. In this study these properties were assessed of cortical and trabecular bone samples from the human femoral neck. In total, 178 trabeculae were obtained from 10 patients suffering a low-trauma fracture and 10 healthy donors (from a previous study) and 141 cortical specimens were newly manufactured from 17 low-trauma fracture patients and 15 controls. Cyclic tensile tests were performed to extract elastic, plastic, viscous, damage, and failure properties with a rheological model. No significant difference of any investigated property was determined. Interestingly, donor aBMD indicated a significant correlation with the post-yield behavior and damage accumulation (modulus degradation) of cortical bone. Cortical bone indicated a significantly larger apparent modulus (17.2 GPa), yield stress (50 MPa), viscosity (17.9 GPas), and damage accumulation (73%), but a decreased toughness (1.6 MJ/m^3^), than trabecular bone (8.8 GPa, 30 MPa, 9.3 GPas, 60%, 3.2 MJ/m^3^, respectively). Qualitatively, cortical bone displayed a linear-elastic phase, followed by a plastic phase with little post-yield hardening. In contrast, trabeculae yielded early, with a pronounced post-yield hardening phase and fractured at larger strains. Only a few correlations between donor mineral status and tissue mechanical behavior were found. It is suggested that the trabecularization of cortical bone with age and disease may not only result in a decreased bone mass, but further causes a transitioning from stiff elastic cortical to soft, viscous trabecular bone. This aspect warrants further investigation to determine its role in age- and osteoporosis-related bone fragility.

**Graphical Abstract:**
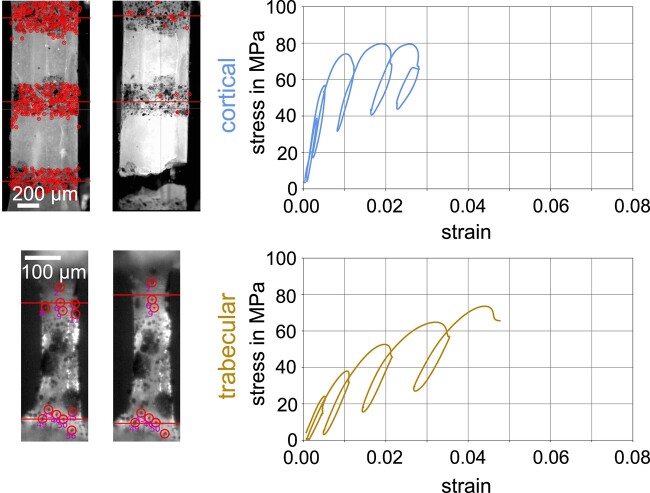

## Introduction

In our aging society, osteoporotic fractures are increasing, eg, it is estimated that the annual number of hip fractures will double during 2018–2050,^1^ whereby especially the increase in the elderly population in Asia will become an economic burden.^2^ The majority are related to primary osteoporosis, which is caused by menopausal estrogen loss (Type I: postmenopausal) and aging (Type II: senile).^3^ Hereby, the incidence of a fracture of the hip in women is almost twice of that of men, because of larger bone loss and risk of fall.^4^ According to the latest European guidance on osteoporosis,^5^ the disease is defined as having a value of bone mineral density (BMD) 2.5 standard deviations (SD) or more below the young female adult mean, assessed by dual X-ray absorptiometry (DXA) at the femoral neck or spine (T score). Fracture risk should be assessed with country-specific FRAX™,^6^ including BMD for patients at intermediate risk. However, a meta-analysis using the FRAX™ 3% 10-yr fracture risk threshold for hip fractures determined a pooled sensitivity (men and women) of only 46% (range: 9%-77%).^7^ Hence, there is a need to increase the detection rate. For instance, trabecular bone score, a tool based on DXA scans to obtain information about the trabecular microarchitecture, has been demonstrated to increase fracture risk assessment (in combination with BMD and FRAX) to a small, but significant extent.^8^

In addition to radiological image analysis, reference point indentation (RPI), an in vivo approach to assess the mechanical properties, was also able to discriminate patients with osteoporosis from healthy subjects.^9–12^ However, as outlined by Jenkins et al.^13^ RPI is a multifactorial measurement (eg, porosity,^13^^,^^14^ tissue mineralization,^14^ microdamage,^13^ and advanced glycation end products^14^ contribute) and does not strongly correlate to a single mechanical property. Hence, it is still unknown if and which mechanical properties of bone are indeed changed in osteoporosis. Further, whole bone models used for fracture risk predication also depend on these values as input parameters. Here, it is essential to be aware, if mechanical properties can be used independently of disease, eg, if only the 3D geometry is altered, or not.

So far, conflicting results have been reported about changes of mechanical properties in osteoporosis. For instance, at the macro-scale (millimeter-sized bone specimens) a reduced elastic modulus and strength in 3-point bending^15^ and tensile tests^16^ of machined cortical bone specimens was reported, but mainly related to increased porosity. Similarly, changed mechanical properties of trabecular bone were linked to a deterioration of the trabecular network.^17^ At the micro-scale (micrometer-sized specimens, level of bone lamellae) mechanical properties of trabecular^18–21^ and cortical^19^^,^^21^^,^^22^ bone tissue were only minorly (mostly not significant) changed in osteoporosis. However, almost no information about the intermediate scale, eg, the tissue scale (level of individual trabeculae and osteons) is available. Limited accessibility, especially of samples from the thin cortical shell of the femoral neck, as well as difficulties in sample preparation and testing might explain this gap.^23^ Determination especially of the failure and damage behavior is essential to discriminate potential differences in diseased bone material eg, during a fall. Hereby, it is important to separately analyze trabecular and cortical bone tissue, since they might be differently affected by osteoporosis and age.^17^ Previously, postmenopausal osteoporosis has been related to a dominant trabecular bone loss in women and senile osteoporosis to cortical and trabecular bone loss in men and women,^24^ requiring also an analysis with respect to sex.

Given the lack of data in the literature, the goal of this study was to perform a thorough mechanical characterization of cortical and trabecular bone tissue from an actual fracture site, ie, the femoral neck of osteoporotic patients and healthy body donors. In principle the same test-approach, as previously applied in tensile tests of individual trabeculae,^25^ was used to assess the complete mechanical behavior using optimization methods to fit the behavior to an extended rheological model.^26^ This approach has the advantage that the following mechanical aspects can be investigated in a single experiment: Elastic modulus (resistance to a reversible deformation), elastic work (completely reversible work, if material is unloaded), viscosity (resistance to a given deformation rate), plasticity (permanent deformation starting after yield point), failure (stress and strain), post-yield work (work that is dissipated and cannot be restored), damage (permanent decrease of modulus due to microdamage in the material). Our hypothesis was that the mechanical behavior of osteoporotic cortical and trabecular bone tissue is different compared with that of healthy control subjects. Separate analysis of both tissues should be performed, as it was further hypothesized that they are differently affected by osteoporosis and, more importantly, that they are two distinct tissues.

## Materials and methods

### Study population and study design

**Figure 1:**
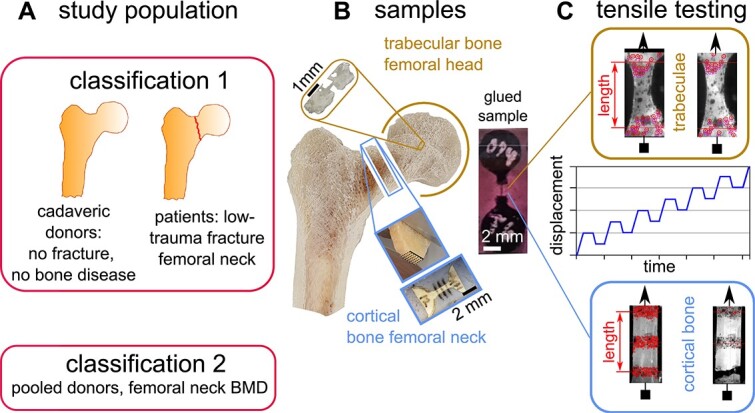
Study flow: A: Study population with classification 1 (low-trauma fracture of femoral neck vs. cadaveric donors without fracture history and free from bone disease) and classification 2 (pooled donors, femoral neck BMD). B: Sample harvesting. Individual trabeculae were obtained from the femoral head, cortical bone specimens were CNC milled from slices of the inferior medial femoral neck. Both tissues were glued into circular end pieces. Tensile testing was performed with an increasing displacement controlled cyclic load-hold-unload protocol. Strain tracking was performed optically.

Femoral head and neck specimens were obtained from a previous study,^10^ whereby full IRB and ethics approvals were obtained for the study (LREC 194/99/1; 210/01; 12/SC/0325) from Southampton and South West Hampshire Research Ethics Committee. In that study^10^ osteoporosis was defined as a low-trauma fracture (group FRAC) that resulted in an intracapsular fracture of the hip (that would not have caused a fracture in individuals with healthy bone). Samples of the control group (CTRL) were obtained from anatomic (cadaveric) body donors, who had no known history of fracture or bone disease. Age (range 57–97), body mass index (BMI), FRAX score, BMD, and T score (both determined with DXA at femoral neck) were also available for both groups (see Table 1; for a detailed information on clinical variables and FRAX calculation see Supplementary Material). Since BMD is still the most widely used classification variable for osteoporosis (and to avoid overlooking of donors with osteoporosis, as classified with BMD in the control group) BMD was used as a continuous, independent variable in a second classification (see Figure 1A). Mechanical tensile tests were performed on individual trabeculae and cortical bone specimens from the same study population, but not necessarily from the same donors. As such, for a direct comparison of trabecular vs cortical bone tissue only a subset of data was used, where both tissue types were available in the same donors. Individual trabeculae have been already tested in a previous study,^25^ whereas cortical bone specimens were newly tested in this study.

**Table 1:** Clinical and osteoporosis factors for patients with low-trauma fracture (FRAC) and healthy donors (CTRL). T score based on femoral neck BMD, determined with DXA. FRAX: fracture risk assessment tool score (without BMD) for trabecular and cortical bone tissue separately. Mean values ± std. *P*: *P*-values determined with Wilcoxon–Mann–Whitney Test (2-sided, unpaired) for all parameters, except sex, where a Fisher exact test was used. F: number of female donors, M: number of male donors.

	**Trabecular**			**Cortical**		
**Parameter**	**CTRL**	**FRAC**	***P***	**CTRL**	**FRAC**	***P***
**Sex, F/M**	5/5	5/5	1.000	12/4	9/6	0.458
**Age, y**	69.5 ± 9.2	74.6 ± 11.0	0.307	68.1 ± 10.2	80.0 ± 9.7	**0.005** ^a^
**BMI, kg/m** ^**2**^	30.1 ± 9.2	26.1 ± 5.2	0.288	29.5 ± 9.1	25.0 ± 4.2	0.272
**T score**	1.12 ± 2.94	−2.41 ± 0.83	**0.002** ^a^	1.53 ± 2.62	−1.75 ± 0.82	**0.011** ^a^
**FRAX, %**	3.1 ± 3.4	13.9 ± 11.3	**0.031** ^a^	3.0 ± 3.2	14.3 ± 8.2	**< 0.001** ^a^

a significant *P*-values (<0.05), marked bold.

### Cortical bone specimens

In total, 32 donors were recruited from the previous study.^10^ In more detail 17 patients sustained a diagnosed low-trauma fracture of the hip (group FRAC: 5 male, 12 female) and 15 body donors were free from any bone-related disease and fracture (group CTRL: 6 male, 9 female). In total, 141 tensile test specimens were prepared from 32 donors (~4.4 specimens per donor). In more detail, 70 specimens were manufactured from 17 patients with osteoporosis and 71 specimens were from 15 control donors.

### Stress–strain data—individual trabeculae

Stress–strain data of individual trabeculae tested in tension were obtained from a previous study.^25^ Hereby, 178 individual trabeculae were successfully tested (89 in each group FRAC and CTRL), obtained from 20 donors. All donors were part of the same original study,^10^ which was used to obtain also cortical samples of the femoral neck. 10 patients were diagnosed with a low-trauma fracture of the hip (group FRAC: 5 male, 5 female) and 10 anatomic body donors free from any bone-related disease and fracture (group CTRL: 5 male, 5 female). These donors were not always the same subjects as those used to obtain the cortical bone samples. However, in a subset of 12 donors 48 cortical and 105 trabecular stress–strain data were available from the same donors. Hence, this subset was used for the direct comparison of mechanical properties of these two tissues.

### Sample preparation

In this study, only cortical bone samples of the femoral neck were newly prepared and tested. The individual trabecula samples were already prepared and tested in a previous study.^25^ Rod-shaped trabeculae were taken from the femoral head and cortical bone from the center of the inferior-medial femoral neck to minimize the risk of mistaking cortical fragments and trabeculae. Further, the ultrastructure was verified with microscopy and μCT, indicating a longitudinal orientation of osteons and lamellae (see Supplementary Material, Figure S1 for details). Individual trabeculae (with residual bone on both ends for attaching end pieces of epoxy glue) were dissected with a hand held miller under a stereo microscope (see Figure 1B and Frank et al.^25^ for details). Dog-bone-shaped specimens were manufactured with a CNC miller from 300 μm-thick bone slices, cut from the inferior-medial femoral neck (see Supplementary Material, Figure S2 for a detailed description). In brief, specimens were manufactured according ASTM D1708-18, “Standard Test Method for Tensile Properties of Plastics by Use of Microtensile Specimens“. Because of the small size and the anatomic shape of the femoral neck, samples had to be rescaled by a factor of 8, resulting in a gauge length of 1.5 mm. Sample storage was done at −80 °C with specimens wrapped in tissue soaked with Hank’s Balanced Salt Solution (HBSS) at pH = 7.4. Also, in between all sample processing steps and testing, samples were stored in HBSS at pH = 7.4. A Speckle pattern was applied to enable optical strain tracking. Subsequently, 3D geometry and tissue mineral density (TMD) of the samples were assessed in HBSS using micro-computed tomography (μCT) with a uCT 100 scanner (Scanco Medical AG, Switzerland) operated at 70 kVp, 200 mAs, average data 3 (each projection recorded 3 times), 4.9 μm voxel size, Al 0.5 mm filter. Prior to 3D imaging of the samples the μCT was calibrated with hydroxyapatite (HA) phantoms to allow quantitative assessment of TMD (each voxel is assessed with a single TMD value). Mean TMD reflects the average TMD per specimen, whereas the standard deviation of TMD indicates the heterogeneity in each specimen. Obtained 3D images were processed using medtool 4.5 (Dr. Pahr Ingenieurs e.U., Austria). In short, reconstructed images were processed with a Gaussian filter (σ = 1, radius = 1) and segmented with a single level threshold of 550 mgHA/cm^3^, whereby unconnected regions of bone and HBSS were removed, using a connected components algorithm. From the segmented data sets bone volume, sample dimensions, TMD, and porosity were calculated. In total 141 cortical bone specimens were successfully manufactured from the inferomedial neck of the femurs. Lastly, samples (trabeculae and cortical specimens) were embedded in circular epoxy end pieces to allow mounting and proper alignment for tensile testing (see Figure 1B).

### Tensile testing and stress–strain evaluation—cortical bone specimens

**Figure 2:**
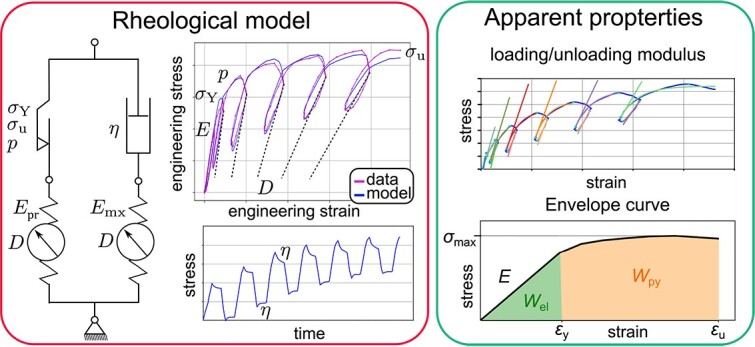
Evaluation of material properties with rheological properties: Long-term modulus E_∞_ = E_pr_: Inclination in the stress–strain curve (resistance to elastic deformation; depending on loading speed it might increase to instantaneous modulus E_0_ = E_pr_ + E_mx_), E_mx_: Visco-elastic modulus, yield stress σ_Y_ (beginning of permanent (plastic) deformation), ultimate stress σ_u_ (failure stress), exponential hardening parameter p (describing shape of stress–strain curve from yield to failure), viscosity η (resistance to a given deformation rate, can be best observed as relaxation of stress in hold phases), damage parameter D (relative decrease of stiffness due to damage, as indicated in stress–strain curve). Apparent properties: Loading and unloading modulus in each cycle (tangent in stress–strain curve), apparent Young’s modulus $\hat{\mathrm{E}\ }$, apparent yield strain $\hat{\mathrm{\varepsilon}}$_y_, apparent ultimate strain $\hat{\mathrm{\varepsilon}}$_u_, maximum stress $\hat{\mathrm{\sigma}}$_max_, elastic work $\hat{\mathrm{W}}$_el_ (area under curve until yield), post yield work $\hat{\mathrm{W}}$_py_ (area under curve until yield), (area under curve from yield to failure).

Tensile testing was performed with a servo-electric load frame (SELmini-001, Thelkin AG, Switzerland), with a nominal step size of 1 μm. Force recording was done with a 100 N load cell (HBM-S2M, Germany, relative error of 0.02% at full scale output). As dehydration of bone samples has a significant effect on obtained material properties,^27^ testing was performed with samples submerged in HBSS (pH = 7.4) at room temperature. A cyclic loading protocol, with increasing load steps and holding times of 10 s in between was used (see Figure 1C), as previously applied for individual trabeculae.^25^^,^^28^ Optical strain tracking was performed with a video camera and a point tracking algorithm (trackpy v.0.5.0^29^). Engineering strain was determined as the relative distance between all particles on the top and bottom line (see Figure 1C, Supplementary Material, Figure S3). Engineering stress was determined by division of recorded force by the mean area, which is simply the obtained bone volume (without pores) from μCT data, divided by sample length (as described previously^30^). Evaluation of apparent mechanical properties (marked with ^: apparent Young’s modulus $\hat{E}$, apparent yield strain ${\hat{\varepsilon}}_y$, apparent ultimate strain ${\hat{\varepsilon}}_u$, maximum stress ${\hat{\sigma}}_{max}$, elastic work ${\hat{W}}_{el}$, post-yield work ${\hat{W}}_{py}$) was performed using the envelope curve and tensile moduli were obtained as tangents in each loading and unloading cycle (see Figure 2—right), as described previously in detail.^25^^,^^28^

### The extended 2-layer rheological model

To obtain further insight into the combined elastic, viscous, plastic, and damage behavior of the cortical and trabecular bone samples, a previously established rheological model from Reisinger et al.^31^ was extended by a damage parameter and utilized to extract material parameters of each sample. This approach already proved to be an effective way to obtain elastic, post-yield, and viscous information from a single mechanical test in previous studies.^25^^,^^28^^,^^31^ Hereby the model was subjected to the strain signal identical to the one from the sample’s experiment. In an optimization procedure, the material parameters were tuned, so that the stress response of the model fitted best to the measured stress response in the experiment (see Figure 2—left). The rheological model itself consists of 2 parallel layers: A Prandtl layer with a linear elastic spring with Youngs’s modulus ${E}_{pr}$ and a plastic slider with yield stress ${\sigma}_Y$, ultimate stress ${\sigma}_u$, and an exponential hardening parameter $p$. The Maxwell layer is built of a second elastic spring ${E}_{mx}$ and a linear damper with viscosity $\eta$. Both spring stiffnesses degrade equally by the damage parameter $D$ which is driven by the amount of equivalent plastic strain. A detailed description of the model’s mathematical framework is provided in Reisinger et al.^26^ For the ease of result interpretation, a long-term modulus is defined as ${E}_{\infty }={E}_{pr}$, an instantaneous modulus as ${E}_0={E}_{pr}+{E}_{mx}$, and a loss factor $\tan \left(\delta \right)$ according to.^31^ The damage at the point of fracture (*D*_end_) and at the point of maximum stress (*D*_max_) was also determined for all successfully evaluated stress–strain curves.

### Statistics

The data were subjected to statistical analysis using RStudio (V. 2022.02.0). Box plots and individual value plots were used to visually inspect the data distribution, variability, and outliers. The Linear Mixed Effects Model (parametric test) was employed to test for differences in groups such as tissue type, ie, trabecular vs cortical, and disease, ie, control vs osteoporotic. The donor was used as the experimental unit. The donor ID was used as a random effect and the groups (tissue type and disease) as a fixed effect. The analysis was performed with the effect of age and gender. Heteroscedasticity was accounted for in the model by allowing different variances per tissue and gender. The serial correlation within each donor (repeated measures) was modeled by the compound symmetry correlation structure. The model assumptions (normality, homoscedasticity) were inspected via the residual plots. The significance level was defined as *P* < .05, and *P*-values *P* > .05 but <.07 were considered tendencies. Evaluation of classification 2 (BMD as continuous independent variable) was performed with scatter plots and calculation of Pearson correlation coefficient (r). Hereby, the median values of the dependent parameters were calculated per donor. Spearman’s rho was used to determine the correlation coefficients between mechanical, material, and tissue mineralization properties (data of specimens, not donors, to determine also nonlinear correlations). For both coefficients, correlations *r* > 0.29 were considered weak, 0.3 < *r* 0.49 moderate, and *r* > 0.5 strong. Coefficient of variation was calculated as standard deviation divided by mean for each mechanical property, individually.

Out of 141 performed tensile tests on cortical bone, 26 tests (13 osteoporotic and cadaveric, each) had to be rejected (18%) from further evaluation, mostly because of a localized deformation outside of the gauge region. This could be linked to porosities and vessels, causing a fracture outside of the gauge region, with an ultimate strain below 1% inside the gauge region. Out of 115 stress–strain curves of cortical bone samples, 85 were successfully evaluated with the rheological model. For individual trabeculae, 31 (17 osteoporotic and 14 cadaveric) tests had to be rejected (17%) from further evaluation, also because of failure outside of the gauge region. Sequentially, 147 out of 178 stress–strain curves were successfully evaluated with the rheological model. As described in previous studies^25^^,^^28^^,^^31^ a strict outlier regimen was used to remove non-physically meaningful values obtained with the rheological model (caused by the optimization approach). As such, an inter quartile range (IQR) test was performed on the root mean squared error (RMSE) of obtained fittings. This caused a removal of 15 trabecular and 10 cortical samples. Afterwards, all mechanical parameters obtained with the rheological model were also subjected to an IQR-test individually, resulting in a further removal of 17 trabecular and 12 cortical samples, on average. Hence, material properties of 115 out of 147 trabecular samples (78%) and 63 out of 115 cortical samples (55%) are reported in the results obtained with the rheological model.

## Results

The stress–strain behavior of low-trauma fracture (group FRAC) and control (group CTRL) samples was obtained from cortical bone samples within the study presented here. Additionally, the stress–strain behavior of FRAC and CRTL samples of individual trabeculae, from donors enrolled in the same study,^25^ was newly evaluated with the described extended rheological model. The fittings of the material properties obtained with the rheological model were significantly (*P* < .05) better for trabecular bone, than for cortical bone (RMSE: (3.2 ± 1.1) MPa vs (4.2 ± 1.5) MPa).

**Figure 3:**
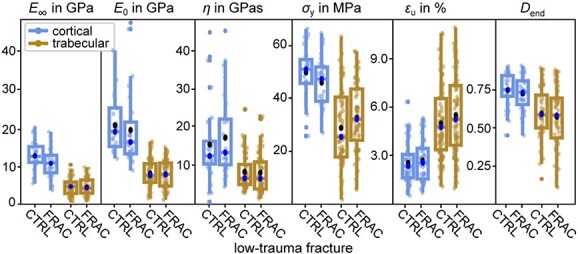
Boxplots for cortical and trabecular bone tissue, with low-trauma fracture as grouping variable. Parameters: Long-term modulus E_∞_, instantaneous modulus E_0_, viscosity η, yield stress σ_Y_, apparent ultimate strain $\hat{\mathrm{\varepsilon}}$_u_, damage parameter at end D_end_. Indicated are data points (bright dots), outliers (solid dots) whiskers (minimum, maximum), box (interquartile range), median (light dot), mean (dark dot).

**Figure 4:**
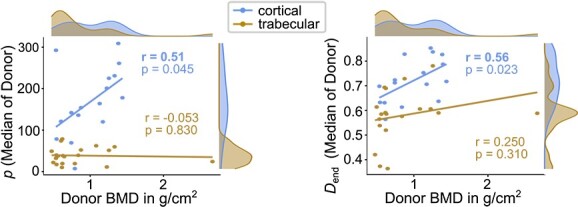
Correlation charts for exponential hardening parameter p and damage parameter at end D_end_. The y-axis demonstrates the median of the parameter of each donor, the x-axis the donor BMD, with marginal histograms for both axes. R: Pearson’s correlation coefficient, separately for cortical and trabecular bone tissue. Strong correlations (r > 0.5) are indicated bold.

None of the investigated rheological material, apparent mechanical, or tissue mineral properties were significantly different between FRAC and CTRL group, neither for trabecular, nor for cortical bone tissue (see Table 2). Corresponding boxplots of selected parameters are visualized in Figure 3. In a second classification, all donors were pooled, and donor BMD was used as the independent, continuous variable (see Figure 4 for selected scatter plots). Hereby, most parameters indicated only a weak (*r* < 0.29) correlation with donor BMD. Strong correlations (*r* > 0.5) were only found for cortical bone, namely positive for exponential hardening parameter and damage at end, and negative for tensile loading modulus at cycle 7 and unloading modulus at cycle 6 (see Supplementary Material, Table S3 for correlation coefficients of all parameters). Interestingly, ultimate stress was moderately negatively correlated with donor BMD for trabecular bone (*r* = –0.44), but only weakly positively for cortical bone.

**Table 2:** Rheological material (top), apparent mechanical (middle), and tissue mineral properties (bottom) for grouping based on low-trauma fracture (FRAC) vs control (CTRL) for trabecular and cortical bone tissue separately. Mean values ± std. *P*: *P*-values determined with a Linear Mixed Effects Model with the effect of age and gender.

	**Trabecular**			**Cortical**		
**Parameter**	**CTRL**	**FRAC**	***P***	**CTRL**	**FRAC**	***P***
*E*_∞_, GPa	4.8 ± 2.3	4.7 ± 2.3	0.977	13.1 ± 4.0	10.9 ± 3.6	0.074
*E*_0_, GPa	8.1 ± 3.5	7.9 ± 3.5	0.957	20.7 ± 7.3	19.4 ± 9.6	0.904
*σ*_y_, MPa	29 ± 15	33 ± 13	0.315	50 ± 10	46 ± 9	0.151
*P*	39 ± 29	41 ± 31	0.585	188 ± 113	153 ± 102	0.176^a^
*σ*_u_, MPa	89 ± 35	97 ± 33	0.620	65 ± 18	61 ± 18	0.561
*η*, GPas	8.5 ± 4.8	8.3 ± 5.3	0.578	15.6 ± 10.7	17.0 ± 9.9	0.252
tan*δ*	0.021 ± 0.014	0.021 ± 0.014	0.504	0.075 ± 0.100	0.069 ± 0.109	0.167
*D*_max_	0.46 ± 0.25	0.46 ± 0.24	0.437	0.58 ± 0.21	0.56 ± 0.19	0.677
*D*_end_	0.60 ± 0.17	0.57 ± 0.19	0.523	0.75 ± 0.11	0.72 ± 0.12	0.377
$\hat{\mathrm{E}}$ , GPa	8.5 ± 5.1	7.7 ± 4.4	0.662	17.2 ± 6.2	15.1 ± 5.7	0.147
${\hat{\mathrm{\varepsilon}}}_{\mathrm{y}}$ , %	0.22 ± 0.16	0.27 ± 0.21	0.413	0.25 ± 0.26	0.28 ± 0.19	0.667^a^
${\hat{\mathrm{\varepsilon}}}_{\mathrm{u}}$ , %	5.0 ± 2.2	5.5 ± 2.4	0.336	2.6 ± 1.3	2.7 ± 1.2	0.699
${\hat{\mathrm{\sigma}}}_{\mathrm{max}}$ , MPa	86 ± 26	89 ± 27	0.663^a^	70 ± 14	73 ± 16	0.919^a^
${\hat{\mathrm{W}}}_{\mathrm{el}}$ , MJ/m^3^	0.017 ± 0.017	0.024 ± 0.022	0.554	0.12 ± 0.19	0.13 ± 0.14	0.707
${\hat{\mathrm{W}}}_{\mathrm{py}}$ , MJ/m^3^	3.1 ± 1.9	3.4 ± 1.9	0.369	1.5 ± 1.1	1.6 ± 0.9	0.805
TMD_mean_, mgHA/cm^3^	962 ± 36	964 ± 35	0.814	1045 ± 23	1037 ± 26	0.163
TMD_std_, mgHA/cm^3^	174 ± 12	174 ± 9	0.725	152 ± 9	150 ± 9	0.572

a Reduced model (low-trauma fracture and sex without age as grouping variable) if original model did not converge.

Gender indicated a significant effect on bone mechanical properties for pooled samples. In cortical bone tissue instantaneous modulus *E*_0_ and elastic work ${\hat{\mathrm{W}}}_{\mathrm{el}}$ were significantly larger, and loss tangent tan*δ* indicated a trend of being larger in males, compared with females (see Table 3). Similarly, in trabecular bone tissue loss tangent was significantly larger in males, while apparent yield strain ${\hat{\mathrm{\varepsilon}}}_{\mathrm{y}}$ showed a trend of being smaller, compared with females. Additionally, the effect of low-trauma fracture was evaluated separately for males and females (see Supplementary Material, Table S1). Hereby, most parameters were not differently affected by gender. However, yield stress was significantly affected only in males and long-term modulus only in females (with a trend of ultimate strain and mean TMD).

**Table 3:** Effect of gender on rheological material (top), apparent mechanical (middle), and tissue mineral properties (bottom) for trabecular and cortical bone tissue. *P*-values determined with a Linear Mixed Effects Model with the effect of low-trauma fracture, age, and gender. This table represents the *P*-value for the effect of gender. The effect of low-trauma fracture is provided in Table 2, and the effect of age in Supplementary Material, Table S2.

	**Trabecular**			**Cortical**		
**Parameter**	**M**	**F**	***P***	**M**	**F**	***P***
*E*_∞_	4.9 ± 2.5	4.6 ± 2.0	0.474	13.0 ± 4.1	11.4 ± 3.8	0.230
*E*_0_	8.1 ± 3.8	7.9 ± 3.2	0.507	24.9 ± 11.2	17.7 ± 5.9	**0.013** ^a^
*σ*_y_	32 ± 14	29 ± 14	0.390	49 ± 9	47 ± 10	0.534
*p*	41 ± 13	39 ± 30	0.513	174 ± 116	167 ± 106	^b^
*σ*_u_	91 ± 13	94 ± 38	0.441	64 ± 19	62 ± 17	0.670
*η*	8.8 ± 4.9	8.0 ± 5.2	0.803	15.4 ± 9.6	17.2 ± 11.1	0.210
tan*δ*	0.022 ± 0.015	0.020 ± 0.013	**0.016** ^a^	0.111 ± 0.130	0.054 ± 0.086	0.061^c^
*D*_max_	0.46 ± 0.25	0.46 ± 0.25	0.463	0.57 ± 0.23	0.57 ± 0.18	0.613
*D*_end_	0.60 ± 0.18	0.57 ± 0.18	0.741	0.75 ± 0.12	0.73 ± 0.11	0.549
$\hat{\mathrm{E}}$	9.0 ± 5.3	7.3 ± 4.1	0.167	17.2 ± 6.9	15.7 ± 5.5	0.271
${\hat{\mathrm{\varepsilon}}}_{\mathrm{y}}$	0.21 ± 0.18	0.28 ± 0.19	0.052^c^	0.29 ± 0.31	0.25 ± 0.17	b
${\hat{\mathrm{\varepsilon}}}_{\mathrm{u}}$	5.1 ± 2.3	5.4 ± 2.3	0.287	2.8 ± 1.4	2.6 ± 1.2	0.145
${\hat{\mathrm{W}}}_{\mathrm{el}}$	0.018 ± 0.020	0.022 ± 0.020	0.145	0.15 ± 0.23	0.11 ± 0.11	**0.047** ^a^
${\hat{\mathrm{W}}}_{\mathrm{py}}$	3.0 ± 1.9	3.4 ± 2.0	0.137	1.6 ± 1.2	1.5 ± 0.9	0.319
TMD_mean_	968 ± 36	958 ± 34	0.310	1042 ± 22	1040 ± 27	0.587
TMD_std_	173 ± 11	176 ± 9	0.457	154 ± 12	149 ± 7	0.101

a significant *P*-values (<0.05), marked bold.

b the model did not converge for these entries.

c *P*-values indicating a tendency (0.05 < *P* < 0.07).

**Figure 5:**
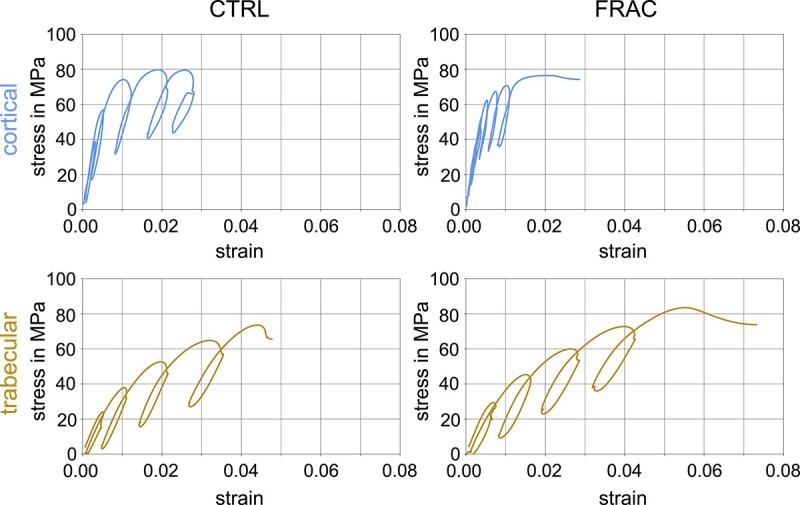
Representative engineering stress–strain curves of cortical and trabecular bone samples. CTRL: Control, FRAC: Low-trauma fracture.

Representative engineering stress–strain curves are presented in Figure 5 (curves were selected to closely match the average material and apparent mechanical properties reported in Table 2). Qualitatively and in terms of stress evolution, cortical bone specimens indicated a pronounced phase at low strain showing almost linear elastic behavior, followed by a less pronounced phase with stress-hardening. In contrast, trabecular bone specimens showed a less pronounced phase at small strains (that did not appear linear) and a much more pronounced post-yield hardening behavior, demonstrating larger toughness compared with cortical bone samples. A direct comparison of trabecular and cortical bone tissue obtained from the same donors indicated a significant difference of all parameters, except loss tangent and yield strain (see Table 4, and Supplementary Material, Figure S4 for boxplots).

**Table 4:** Rheological material (top), apparent mechanical (middle), and tissue mineral properties (bottom) for grouping based on tissue type (trabecular vs cortical) for a subset of identical donors. Mean values ± std. *P*: *P*-values determined with a Linear Mixed Effects Model with the effect of age and gender.

**Parameter**	**Trabecular**	**Cortical**	***P***
*E*_∞_, GPa	4.9 ± 2.3	13.7 ± 3.5	**<.001** ^a^
*E*_0_, GPa	8.3 ± 3.5	23.0 ± 9.5	**<.001** ^a^
*σ*_y_, MPa	30 ± 15	50 ± 9	**<.001** ^a^ ^,^ ^b^
*p*	41 ± 30	184 ± 115	**<.001** ^a^
*σ*_u_, MPa	91 ± 35	68 ± 16	**<.001** ^a^
*η*, GPas	9.3 ± 5.1	17.9 ± 10.9	**<.001** ^a^
tan*δ*	0.021 ± 0.014	0.070 ± 0.087	.059^c^
*D*_max_	0.46 ± 0.26	0.61 ± 0.19	**.005** ^a^
*D*_end_	0.60 ± 0.18	0.73 ± 0.12	**.004** ^a^
$\hat{\mathrm{E}}$ , GPa	8.8 ± 5.0	17.2 ± 6.1	**<.001** ^a^
${\hat{\mathrm{\varepsilon}}}_{\mathrm{y}}$ , %	0.22 ± 0.16	0.25 ± 0.28	.870
${\hat{\mathrm{\varepsilon}}}_{\mathrm{u}}$ , %	5.1 ± 2.2	2.6 ± 1.3	**<.001** ^a^
${\hat{\mathrm{\sigma}}}_{\mathrm{max}}$ , MPa	88 ± 27	73 ± 15	**<.001** ^a^ ^,^ ^b^
${\hat{\mathrm{W}}}_{\mathrm{el}}$ , MJ/m^3^	0.017 ± 0.017	0.128 ± 0.215	**.005** ^a^
${\hat{\mathrm{W}}}_{\mathrm{py}}$ , MJ/m^3^	3.2 ± 1.9	1.6 ± 1.2	**<.001** ^a^
TMD_mean_, mgHA/cm^3^	966 ± 37	1040 ± 21	**<.001** ^a^
TMD_std_, mgHA/cm^3^	175 ± 10	151 ± 10	**<.001** ^a^

a Significant *P*-values (<.05), marked bold.

b Reduced model (low-trauma fracture and sex without age as grouping variable) if original model did not converge.

c *P*-values indicating a tendency (.05 < *P* < .07).

**Figure 6:**
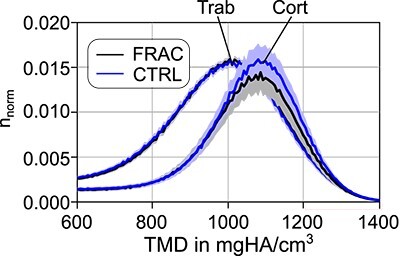
Normalized histogram for tissue mineral density (TMD) for trabecular (Trab) and cortical (Cort) samples (all TMD values of all test specimens are included), grouped for low-trauma fracture (FRAC) and control (CTRL).

Mean and standard deviation of TMD was not significantly different between fracture and control groups, neither for trabecular bone samples, nor for cortical ones (see Figure 6 and Table 2). However, mean TMD was significantly (*P* < .001) larger and standard deviation (std) was significantly smaller for cortical bone compared with trabecular samples (see Table 4).

**Figure 7:**
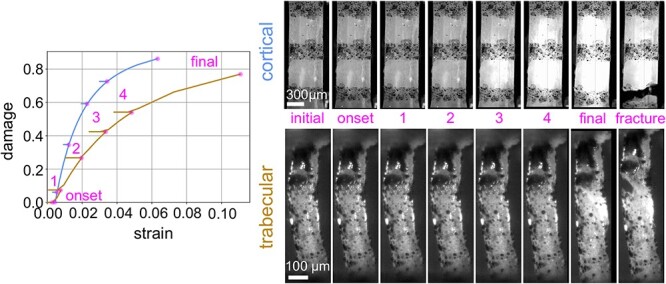
Evolution of damage for a representative cortical and trabecular bone sample. Images are given for the initial (start) point, the onset of damage, the last point of each loading cycle, the final point before fracture, and after fracture. Left: Quantitative increase of damage, obtained with the rheological model, with increasing strain. Right: Qualitatively the image series indicated an increase of the white appearing area.

**Figure 8:**
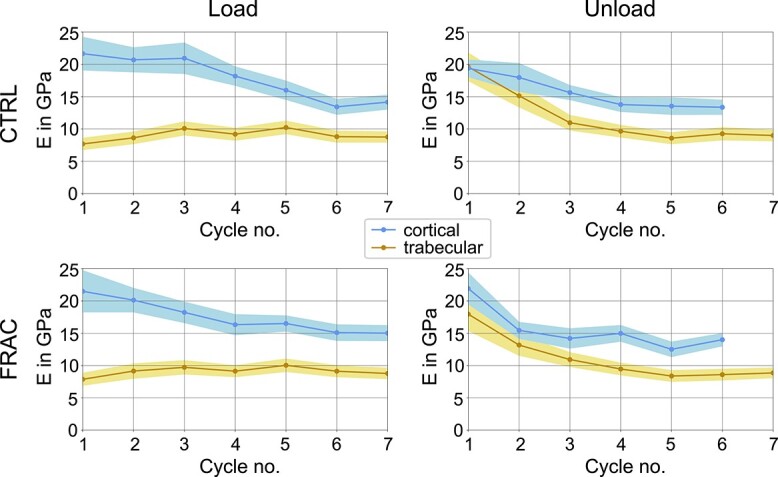
Tensile modulus in each loading and unloading phase, shown as mean value with 95% confidence interval. CTRL: Control, FRAC: Low-trauma fracture, for cortical and trabecular bone separately.

Further, the evolution of damage was different between cortical and trabecular bone tissue samples (see Figure 7). Qualitatively (relative white area in image series) and quantitatively (rheological damage parameter in diagram), cortical bone samples accumulated damage more rapidly and to a larger extent than individual trabeculae. Hereby, the effect of optical whitening of bone has been previously linked to microdamage accumulation.^32^ Neither loading nor unloading modulus was different between control and low-trauma fracture specimens, in any tissue. Loading modulus was significantly larger for cortical bone specimens, compared with individual trabeculae, in all loading cycles (see Figure 8). In contrast, unloading modulus was not significantly different between the two tissues in the first 2 cycles, but in cycles 3 to 6. Interestingly, loading modulus and unloading modulus decreased on average (last cycle in all 4 groups) to 14.1 GPa for cortical bone. In individual trabeculae, the unloading modulus decreased to 8.9 GPa on average (last cycle of both groups), whereas loading modulus of trabecular bone stayed almost constant around 9.1 GPa (on average for all cycles).

Coefficient of variation (CV: relation of standard deviation to mean) of mechanical properties ranged from 0.16 to 0.74 for cortical and trabecular bone, except for loss tangent of cortical bone (1.45), yield strain of trabecular and cortical bone (0.78 and 0.86), and elastic work of trabecular and cortical bone (0.98 and 1.32). Detailed CV values are provided in Supplementary Material, Table S4 for all parameters. Post-yield work showed a strong negative correlation with mean TMD (*r*_s_ = −0.54, *P* < .001), and a strong positive one with the standard deviation of TMD (*r*_s_ = 0.35, *P* < .001) both for pooled data. In contrast, mean TMD indicated a strong positive correlation with apparent modulus, storage modulus, and instantaneous modulus (*r*_s_ ~ 0.6, *P* < .001, on average for pooled data).

## Discussion

In this comparative study, a tensile testing approach was developed to obtain the mechanical properties of sub-millimeter-sized bone specimens from of the thin cortical shell of the human femoral neck, for the first time. Previously, only Gastaldi et al.^23^ successfully determined the mechanical properties at this region in 3-point bending tests of 0.4 mm-thick micro-specimens. The majority of previous studies^15^^,^^33–37^ determined the mechanical properties in millimeter-sized specimens of the diaphysis of femora, most likely due to easier sample harvesting procedures. However, it has been demonstrated that there is a substantial difference of the mechanical properties between the femoral neck and shaft, both in nanoindentation experiments^38^ and scanning acoustic microscopy.^39^ A direct comparison of the obtained parameters in this study with respect to previous literature is thus not possible. Hence, checking the validity of obtained mechanical values could be only performed with previous literature on the femoral diaphysis. Hereby, there was a good agreement between the determined values in this study and previous literature for tensile elastic (loading) modulus of longitudinal specimens,^34^^,^^40–42^ instantaneous modulus^33^^,^^34^^,^^43–45^, and loss tangent.^33^^,^^43–45^ A more detailed discussion of those values is provided in the Supplementary Material. Tensile yield strain was previously reported ranging from 0.39 to 0.72%^37^^,^^40^^,^^42^^,^^46^ and tensile yield stress as 72,^37^ 122^42^, and 129 MPa.^40^ In this study, the average apparent yield strain was 0.25% and the yield stress 50 MPa. These comparatively much lower values might be attributed to the smaller sample thickness (~2 mm vs 0.3 mm here) and hence a more pronounced sensitivity to local yielding effects. Similarly, previously reported ultimate stress was also much larger (93 to 165 MPa)^37^^,^^40–42^^,^^46^ than the determined 65 MPa in the study presented here. Ultimate strain was reported to range from 1.5% to 3.1%,^37^^,^^40–42^^,^^46^ well matching with the 2.6% reported here. Further, post-yield work was previously determined as 1.5 MJ/m^3,^^37^ the same value as reported here. However, also 3.2 MJ/m^3^ were reported previously,^42^ likely related to an almost 2-fold failure stress and a younger donor (35 yr old). Apparently, failure strain is not influenced by the smaller sample size, whereas yield onset and failure stress are lowered, probably due to a more pronounced flaw sensitivity. Further, Abdel-Wahab et al.^33^ determined a large anatomical variation of failure stress in the human femoral diaphysis, eg, 65 MPa in the lateral and 118 MPa in the anterior region. The authors linked this difference to a different microstructure of the cortex along the circumferential direction, with different osteon configurations. Similarly, Malo et al.^39^ demonstrated a large variation of microstructure and elastic properties in the femoral neck and diaphysis. Hence, one can speculate that similarly also differences for yield and failure properties between femoral neck and diaphysis exist.

Similar to previous studies on the fatigue behavior of cortical bone,^40^^,^^46^ we observed a gradual decrease of tensile modulus with increasing cyclic strain. The final amount of accumulated damage was previously reported as 0.71,^46^ which is well between the values of 0.58 for damage at maximum stress (*D_max_*) and 0.75 for Damage at failure (*D_end_*), as reported here. In previous loading–unloading experiments, unloading modulus was reported contrary as being larger^33^ or smaller^47^ than the initial loading modulus. In this study no significant difference was found between the first loading and unloading cycle for cortical bone tissue (see Figure 8 in manuscript). Unloading modulus already decreased in cycle 2 (FRAC) or 3 (CTRL), whereas loading modulus indicated a pronounced decrease only starting in cycles 4–5 and stayed almost constant in subsequent cycles. Similarly, Nyman et al.^46^ reported a sharp drop in the elastic modulus after the yield point with a reduced decrease afterwards. Interestingly, Joo et al.^47^ also reported that loading modulus was not effected after a damage cycle (under zero-strain hold), whereas unloading modulus was. However, in that study both moduli were affected after a damage cycle at zero-stress hold, demonstrating that the representation of damage depends on the chosen test condition.

In summary, our presented results showed an overall good agreement with current literature. Hence, the presented methodology can be seen as a reliable test for characterization of the mechanical properties of the thin cortical shell of the femoral neck.

### Effect of osteoporosis

No significant difference of any material or mechanical property between control and low-trauma fracture donors was observed, neither for cortical nor for trabecular bone tissue. This is in accordance with previous studies that did not detect a difference in elastic modulus or hardness in nanoindentation experiments on cortical bone of human femoral necks^22^ or trans iliac core biopsies.^19^ Further, Vennin et al.^21^ also found no significant difference of dynamic material properties (storage and loss modulus) in trans iliac core biopsies (a non-load bearing site) of osteoporotic and control donors. However, they determined small (9%-12%), but significant, lower median values for elastic modulus and hardness of cortical bone in the osteoporotic group. Based on a smaller within-specimen variability of those 2 parameters in osteoporotic donors, they assumed that a lower mechanical heterogeneity of bone tissue contributes to a decreased fracture resistance. In this study, material toughness and damage accumulation were directly assessed at the femoral neck (a major load bearing site), but no significant difference was observed between control and low-trauma fracture donors. Additionally, intra-donor variation (within an individual donor) of mechanical and material properties was assessed and put in relation to grouping based on a low-trauma fracture (see Supplementary Material, Table S5). Donors sustaining a low-trauma fracture did not demonstrate a lower tissue heterogeneity (except yield stress of trabecular bone), as suggested by Vennin et al.^21^

In a second classification all donors were grouped, and BMD was used as the independent variable to determine the correlation with obtained parameters. Interestingly, the hardening exponent of cortical bone indicated a strong positive correlation, meaning that the increase of stress after the yield point is differently affected in patients with low BMD, compared with those with high BMD. Further, damage at end (percentage of modulus degradation before fracture) showed a strong positive correlation with BMD for cortical bone, an indication that bone of patients with larger BMD is able accumulate more damage before fracturing (see Figure 4). Accordingly, the low-fracture group indicated a more rapid decrease of unloading modulus of cortical bone (see Figure 8, cycle 2), meaning that damage accumulation might occur faster than in controls. The degradation of elastic modulus has been previously related to an increase of microscopic microdamage accumulation.^48^ Hence, there is an indication that microdamage formation might be different in osteoporosis of cortical bone. One might speculate that initially (ahead of testing) more microdamage was present in osteoporotic bone, causing an earlier degradation of modulus. However, this has not been directly assessed in this study and previously, an increase of the amount of microdamage has only been determined with age, but not with osteoporosis.^48^ Although most investigated parameters displayed only a weak correlation with donor BMD, at least the post-yield and damage behavior of cortical bone tissue seems to be altered with decreasing BMD, whereas trabecular bone is not affected to that extent. Hence, it is assumed that the changes of mechanical properties of bone tissue are only a minor contributor to the increased fracture risk in osteoporosis. In this context, other measures, such as volumetric bone quantity and porosity may have a larger influence.

In the context of porosity, previous studies on millimeter-sized cortical bone specimens detected a significantly reduced elastic modulus,^15^^,^^16^ yield stress,^15^ failure stress,^15^^,^^16^ and post-yield work^16^ in donors with osteoporosis. However, cortical porosity was significantly higher in osteoporotic donors in both studies,^15^^,^^16^ indicating a structural influence of obtained material properties. Accordingly, Dong et al.^49^ determined a significant correlation of longitudinal elastic modulus of cortical bone with porosity. In a linear regression model, they demonstrated that the determined elastic modulus of 16.6 GPa increases to 21.4 GPa, if porosity is considered. This might also explain the difference in obtained values for elastic modulus, depending on whether porosity was considered or not.

The classification based on the occurrence of an osteoporotic fracture did not show any significant differences in the mechanical properties, in accordance with our previous study on individual trabeculae.^25^ However, using patient BMD as a continuous independent variable demonstrated that bone obtained from patients with a larger BMD was able accumulate more damage before fracturing. Hence, not only the bone mass, but also the bone material quality itself might be at least minorly reduced in patients with low BMD.

### Cortical vs trabecular bone tissue

Previous studies showed that trabecular bone tissue is less stiff than cortical bone tissue via nanoindentation^50–53^ and 3-point bending^54–56^ experiments, meaning that cortical bone tissue is not simply dense trabecular bone,^57^ as originally suggested by Wolff.^58^ However, tensile properties have only been directly compared in one study,^57^ with an elastic modulus of 10.4 GPa for trabecular and 18.6 GPa for cortical bone tissue, in good agreement with the determined 8.8 and 17.2 GPa (apparent tensile moduli) in this study. However, this study is the first one to directly compare the tensile mechanical properties of both tissues at the femoral neck and head (from the same donors). This is important, since a large anatomical variation of obtained elastic moduli between the femoral diaphysis and neck has been reported previously.^38^^,^^39^ As such, this study uncovered not only significant differences in the elastic, but also in the viscous, yield, post-yield, damage, and failure properties between neighboring cortical and trabecular bone (see Table 4). Figure 5 illustrates that the stress–strain behavior of cortical bone tissue shows an almost linear-elastic phase, followed by linear hardening, with little increase of post-yield stress. Nevertheless, the rheological model also associates cortical bone with viscous behavior. In contrast, trabecular bone tissue shows early yielding with a pronounced exponential post-yield hardening and a significantly larger post-yield work.

Most previous studies^50^^,^^52–54^^,^^56^ focused on differences in the elastic properties of trabecular and cortical bone tissue. However, a direct comparison of elastic moduli obtained from ultrasonic experiments to micro tensile experiments demonstrated a strain rate dependency of both tissues.^57^ In this study, dynamic mechanical properties were directly assessed. Hereby, storage modulus and viscosity of trabecular bone tissue were significantly lower than that of cortical bone, and loss tangent showed a trend of being lower in trabecular bone. This is in agreement with Isaksson et al.,^51^ who also found a significantly lower storage modulus and viscosity of trabecular bone tissue in nanoindentation experiments, and loss tangent was partly affected (depending on the chosen test protocol). In course of this study, damage accumulation was assessed in terms of reduction of elastic modulus in loading and unloading phases, as well as with the rheological model. Loading modulus of cortical bone tissue was significantly larger than trabecular bone tissue in all cycles. Further, it decreased with increasing load cycle, in contrast to trabecular bone. This could be potentially related to the observation that cortical bone tissue accumulated significantly more damage (a larger relative degradation of modulus) than trabecular one (damage at end: 0.73 vs 0.60; see Figure 7 for representative samples). Individual trabeculae sustained a significantly larger ultimate strain and stress than cortical bone tissue. Hence, post-yield work was approximately 2 times larger (3.2 MJ/m^3^ vs 1.6 MJ/m^3^). Accordingly, ultimate strain was reported to be significantly larger in individual trabeculae,^55^ compared with cortical bone. Further, fatigue and failure behavior was also reported as being significantly different between machined trabecular and cortical bone tissue specimens.^54^ In accordance with those and our findings, intra-donor variability of mechanical and material properties (variation within an individual donor) was significantly lower in cortical bone tissue than in trabecular bone (see Supplementary Material, Table S6). Put in another way, cortical bone tissue is less heterogeneous, which likely contributes to the lower toughness and energy absorption, compared with trabecular bone.

In addition to the described differences in the mechanical behavior, also TMD of individual trabeculae was significantly lower than that of cortical bone specimens, in accordance with previous studies about bone mineral density distribution,^59^ ash fraction,^60^ and mineral to matrix ratio.^55^ TMD was strongly positively correlated with elastic modulus and negatively with post-yield work (toughness), indicating its importance in determining the mechanical properties of bone. Similarly, the smaller variation of TMD in cortical bone tissue could also be related to the decreased toughness, in accordance with Vennin et al.^21^ Previous studies determined a significantly lower water^60^^,^^61^ and organic^60^ fraction in cortical bone tissue, which might further explain the reported differences in the mechanical properties here. However, this has not been assessed in this study and should be addressed in future.

Previously, postmenopausal osteoporosis in women has been related to trabecular bone loss and senile osteoporosis (in both men and women) to cortical and trabecular bone loss.^24^ Hence, in this study the effect of low-trauma fracture was also evaluated separately for males and females (see Table S1). Hereby, most parameters were not differently affected by sex, except yield stress in males only, and long-term modulus in females only. In accordance, a direct comparison of males vs females indicated no significant effect of most investigated mechanical and material properties (see Table 3). However, for cortical bone tissue instantaneous elastic modulus and apparent elastic work were significantly larger, and loss tangent tended to being larger in males than females. In accordance, Wu et al. also found significant different time constants with respect to gender, whereas elastic modulus was not affected.^35^ Hence, the effect of gender on the mechanical properties of cortical bone might only be present at sufficient large loading rates. This is of great interest since the lower instantaneous resistance of cortical bone in females could be a potential factor in the greater likelihood of an osteoporotic fracture. But the number of donors in this study was too low to draw such a general conclusion and further investigation is necessary for verification. In trabecular bone tissue there was also mostly no effect of gender onto mechanical or material properties, except of a significantly larger loss tangent in males and a trend of a smaller apparent yield strain in males (see Table 3). Hence, individual trabeculae of males tend to yield earlier and exhibit a significantly different dynamic behavior than those of females. Again, a larger number of donors is necessary to determine clinically relevant effects of these findings.

Age also indicated a significant effect onto a few investigated mechanical and material properties, while most parameters were not affected (see Supplementary Material, Table S2 for a further discussion about the effect of age and tissue mineralization).

### Increased fracture risk related to material transition from cortical to trabecular bone?

In this study no detectable changes of material properties of cortical or trabecular bone tissue were observed with respect to low-trauma fracture and only a few parameters were affected with respect to donor BMD. Hence, one might argue that consequently such changes do not contribute much to fracture risk. This statement is flawed, however, as it is clear, from this and other studies,^38^^,^^50–52^^,^^54–57^^,^^62^^,^^63^ that trabecular and cortical bone are two structurally and mechanically different tissue types. Consequently, the structural remodeling of the femoral neck with age and diseases, as described in a commentary by Zebaze and Seeman,^64^ should be considered. In particular, they comment on a highly porous phase of cortical bone, spatially transitioning into trabecular bone. This transitional zone area was determined to be significantly increased in post-menopausal women sustaining non-vertebral fractures.^65^ From a mechanical standpoint, this suggests a gradual change from cortical to trabecular bone tissue structure and possibly also material properties. For example, Kim et al.^66^ determined different nanoindentation parameters of periosteal, endosteal, and trabecular bone tissue. With the knowledge of the significant differences between trabecular and cortical bone material properties at the femoral neck, as also detailed in this study, the type of tissue and the composition of tissues can have large influence on the overall mechanics, the fracture toughness, and fracture risk. Integrating such knowledge into 3D imaging diagnostics, better predictions of bone fragility may already be possible. In addition, this also shows that tissue material properties may still have a large influence on clinical fracture risk, not in a change of properties within one tissue but via transitioning of specific sub-volumes from cortical to trabecular bone.

In this context, a significant reduction of cortical bone and increase of trabecular bone at the femoral neck would firstly reduce the compressive and flexural modulus, lower the overall yield point, and likely lead to increased damage accumulation followed by a remodeling response. The latter might then worsen the situation even further, by producing even more transitional or trabecular bone and reducing the amount of cortical bone.

### Limitations

A major limitation was that the number of donors per group was relatively small, especially for the males. To also provide enough specimens per donor (given the known anatomical variation), a trade-off was made between the number of specimens and donors. The whole procedure of harvesting, preparation, and testing this sub-millimeter-sized specimens is very tedious and requires a lot of expertise, meaning that a total number of 178 trabeculae and 141 cortical specimens was large, in comparison with the number of specimens at this scale in previous studies. Still, a larger number of donors might be required to draw a more general conclusion. A further limitation was that cortical specimens of the fracture group were slightly, but significantly, larger than control ones. A possible reason might be that there was less trabecular bone present for clamping in fracture cases. However, Gastaldi et al.^23^ argued that the size-dependency is likely smaller in the femoral neck, compared with the diaphysis. Further, the rheological model used is descriptive, using the minimum number of necessary elements for sufficiently modeling the mechanical behavior of bone tissue. As such, no direct relation to underlying physical contributions, eg, different time scales of viscous contributions, could be gained. Previously, a fast and a slow viscoelastic component was suggested for cortical bone tissue,^67^^,^^68^ related to collagen and the anisotropy of cortical bone, meaning that this effect was not accounted here. Moreover, the coefficient of variation of mechanical properties ranged from 0.16 to 0.86, except for loss tangent and elastic work see Supplementary Material, Table S4 for details. This could be related to the optimization process of the rheological model (optimization of several properties at once might cause non-optimal fits in non-sensitive parameters^31^) and a large biological variation.^66^^,^^69^ However, previously coefficient of variation of nanoindentation protocols for measurement of viscoelasticity was also reported ranging from 0.09 to 0.40.^51^ Another limitation could be the use of cortical fragments, instead of individual trabeculae. Using light microscopy and μCT, it could be demonstrated that there was a substantial difference in the ultra-structure between the 2 groups (see Supplementary Material, Figure S1). Here, chemical analysis could be used additionally to validate this assumption even better. TMD was assessed with μCT calibrated with hydroxyapatite up to 800 mgHA/cm^3^. However, Mashiatulla et al.,^70^ demonstrated that obtaining correct absolute mean TMD values, resembling those obtained with qBEI, requires calibration with phantoms up to 1860 mgHA/cm^3^, limiting the obtained TMD values of this study. However, it is assumed that relative differences between osteoporotic and control specimens should have still been captured. An additional limitation was that, although care was taken to dissect individual trabeculae in our previous study^25^ from the corresponding similar locations in the femoral head, there could be a potential bias of specimen selection, due to the increased porosity in osteoporotic femoral heads. Three out of 16 donors of the cortical group (19%) and 1 out of 10 donors of the trabecular group (10%) took medication for osteoporosis (alendronic acid), potentially affecting material properties. However, as the effect of osteoporosis was more pronounced in cortical bone tissue, and since the relative number was low, it is speculated that this effect did not contribute a lot to our findings.

## Conclusion

The material and mechanical properties of cortical and trabecular bone tissue of the proximal femur are not significantly changed in patients sustaining a low-trauma fracture, but minorly altered in patients with low BMD, at least for cortical bone. Trabecular and cortical bone tissue at the femoral neck are tissues with significantly different material properties, and differently affected by osteoporosis. Individual trabeculae show early yielding with a pronounced post-yield hardening phase and a large toughness. In contrast, cortical bone tissue of the femoral neck demonstrates a linear-elastic phase, with little post-yield hardening and approximately half the toughness of individual trabeculae. Given that aging and osteoporosis causes trabecularization at the endosteal surface, whole bone mechanical properties might not be only weakened by a structural change, but additionally by local changes in material properties. However, this should be directly investigated in a future, multi-scale study.

## Supplementary Material

Supplementary_firstlook_ziae049

## Data Availability

The data that support the findings of this study are openly available in Frank, Martin (2023), “BoneMechDamage”, Mendeley Data, V1, doi: 10.17632/hdpmjgx8rg.1.
