# Supplementary material for: Cortical and trabecular mechanical properties in the femoral neck vary differently with changes in bone mineral density
Source: JBMR Plus. 2024 Apr 9;8(6):ziae049. doi: 10.1093/jbmrpl/ziae049 (PMC11088358; doi:10.1093/jbmrpl/ziae049)
Supplement: Supplementary_firstlook_ziae049 [file supplementary_firstlook_ziae049.docx]

# **Supplementary**

## **Study population details**

Clinical information about donors was obtained from Jenkins (1) and included: “Relevant medical conditions (diabetes, thyroid disease, hyperparathyroidism, avascular necrosis, Paget’s disease, hypogonadism, osteogenesis imperfect, chronic malnutrition, rheumatoid arthritis, osteoporosis and osteoarthritis), medical history (fracture history and hysterectomy), medication (antiresorptives, steroid use and hormone replacement therapy), lifestyle factors (current smoking, units of daily alcohol consumption) and other factors relevant to bone health (height, weight and familial osteoporosis) for all groups.” This data was collected through researcher-led, self-assessment questionnaires and access to the participants’ medical notes for the low-trauma fracture group. For the control group, these data were provided by the tissue bank. Hence, Jenkins (1) calculated the FRAX score for all donors using the online fracture risk assessment tool to predict 10 year fracture probability for the hip or any major osteoporotic fracture: “The tool was used with clinical factors alone (age, sex, height, weight and binary responses to the following: previous hip fracture, parent hip fracture, rheumatoid arthritis, glucocorticoid use, current smoker, greater than 3 units of alcohol per day and secondary osteoporosis) and in combination with the femoral neck BMD.” Calcium and vitamin D levels for the osteoporotic group were collected where possible, with average values of (2.32 ± 0.11) mmol/l and (60.8 ± 42.6) nmol/l, respectively. Hence, the values lie within the physiological range of 2.1 to 2.6 mmol/l for calcium > 50 nmol/l for vitamin D.

## **Ultrastructure of individual trabeculae and cortical bone**

To ensure that trabeculae and cortical bone tissue were indeed two distinct tissues, their ultrastructure was analyzed with light microscopy and µCT (see Figure S1). It was demonstrated that cortical bone samples showed osteons oriented longitudinally with clearly detectable cement lines running parallel to the longitudinal axis. Further, µCT images indicated vascular cavities, which were also mostly oriented longitudinally with an average porosity of 2,5%. In contrast, no clear cement lines, cavities, or other longitudinally oriented structures were visible in light microscopy or µCT images of individual trabeculae. Hence, the absence of these structures in all samples indicates that indeed two distinct tissue types were analyzed. As such, the risk that cortical fragments were taken instead of trabeculae was very unlikely. Tissue mineral density from cortical bone taken farther away from the central region of the femoral neck (closer to the endocortex or periost) indicated no trend of decreased mineral density. Thus, it was assumed that there was no relevant change in material properties across the radial direction, at least at the investigated scale.


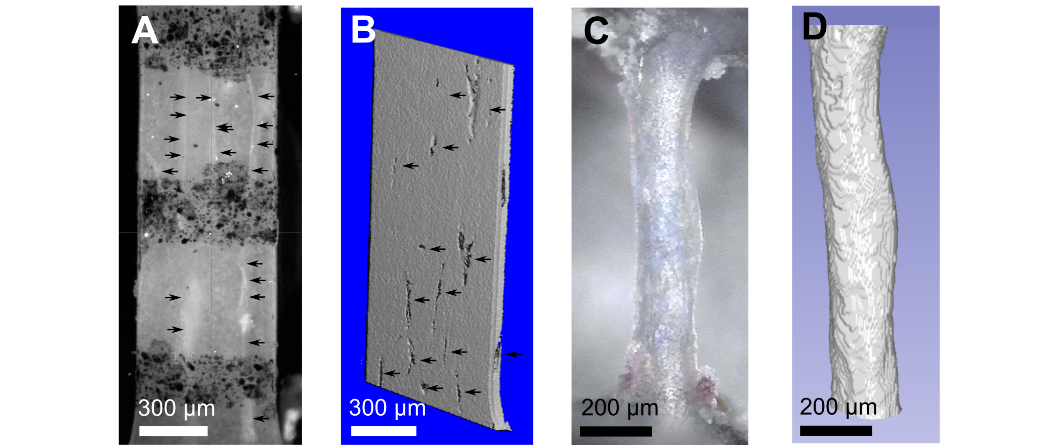


Figure S1: Ultra-structure of bone specimens. A: microscopy image of cortical bone specimen, with cement lines appearing bright (marked with arrows). B: µCT image of cortical bone specimen, with small vascular structures marked with arrows. C: polarization microscopy image of individual trabeculae, without any evident microstructure. D: µCT image of individual trabeculae, without detectable vascular structures.

## **Sample preparation details**

In a pre-study, the femoral neck, and the femoral shaft of one control donor were used to assess the optimal scaling of the used sample geometry, since the average width of the sliced femoral necks (obtained from a previous study (2)) was ~ 8 mm. As the required center length of ASTM D1708 – 18 is 12 mm, re-scaling with a factor of 2.5, 3, 5, and 8 was done, e.g. new length was 1.5 mm for scaling factor 8. Design of sample geometry was done in Inventor (2021, Autodesk, Inc, U.S.), whereby the clamping region was modified to enable fitting into sample holders with ⌀ 5 mm, as previously used for tensile testing of individual trabeculae (3). Samples were manufactured and tested in tensile mode as described in the next paragraphs. Only samples rescaled by a factor of 8 ensured, that specimens fractured inside the gauge region. Larger samples fractured mostly in the clamping region and thus, could not be used. Sample storage was done at –80°C with specimens wrapped in tissue soaked with Hank’s Balanced Salt Solution (HBSS) at pH = 7.4. Also, in between all sample processing steps and testing, samples were stored in HBSS at pH = 7.4.

As shown in Figure S2 300 µm-thick bone slices were cut from the inferomedial region of femoral neck sections using a low-speed saw (Isomet, Buehler, ITW Test & Measurement GmbH, Germany). Cutting was performed along the longitudinal axis of the femoral neck (and thus along osteonal orientation), preferentially at the thickest location of the cortex. Then, the contour for CNC milling was outlined with a marker under a stereomicroscope (SZX10, Olympus Corporation, Japan), ensuring a longitudinal alignment of the osteons and avoiding large pores, eg. Haversian or Volkmann’s channels, inside the specimen. For that a 3D printed specimen template was used (Prusa Research Original Prusa SL1, slice thickness 25 µm, initial layer 17s, hardening time 7.5 s). Bone slices were mounted onto a CNC router (BZT PFK-0203-PX, BZT Maschinenbau GmbH, Germany) and aligned on a mounting station, according to the marked outline. This step was essential to ensure that the specimen is milled out at the position outlined. Milling was performed with a dental driller (NeoDiamond FG, form 108, cylinder - ISO 010, Henry Schein Dental Austria GmbH, Austria), with specimens submerged in distilled water. Milled specimens were cleaned from shavings with tweezers and a scalpel, under a stereo microscope. Next, each specimen was put in a 3D printed sample holder, to enable application of a speckle pattern for optical strain tracking. For this, masks with three 0.4 mm slits, separated by 0.75 mm were designed and 3D printed. The masks were placed onto the bone specimens, located inside the 3D printed sample holders, and fixed with 3D printed lids. This procedure ensured accurate application of the speckle pattern. Specimens inside 3D printed samples holders were sprayed with Black spray paint (Dupli-Color Lackspray Aerosol-Art RAL 9005, deep black, European Aerosols GmbH, Germany).


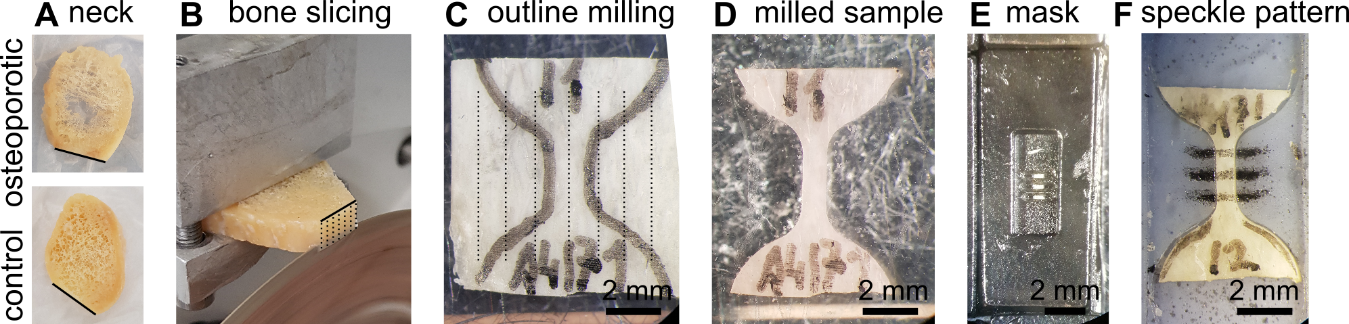


Figure S2: Sample preparation. Bone pieces of femoral neck (A) were sliced with a low-speed saw into 300 µm thick slices (B). The contour was outlined to avoid pores inside the specimen and to ensure longitudinal alignment of osteons (C). Specimens were CNC milled (D) and a speckle pattern was applied with a 3D printed mask (E) for optical strain tracking (final sample, F).

## **Sample testing details**

Samples were mounted by putting the circular 5 mm mounting regions inside the corresponding holes of custom-made sample holders (see Figure S3). Since the holes had a depth of 2 mm, 3D printed circular spacers of 0.85 mm thickness were put underneath and above the 0.3 mm-thick bone specimen to ensure alignment along the central machine axis. The circular shape was chosen to enable self-alignment of samples during application of the pre-load (0.5 N). Then, 2 mm aluminum plates were put on top of the spacer and fixated with a transistor fitting spring (THFM 1, Fischer Elektronik GmbH, Austria), to avoid out of plane movement.


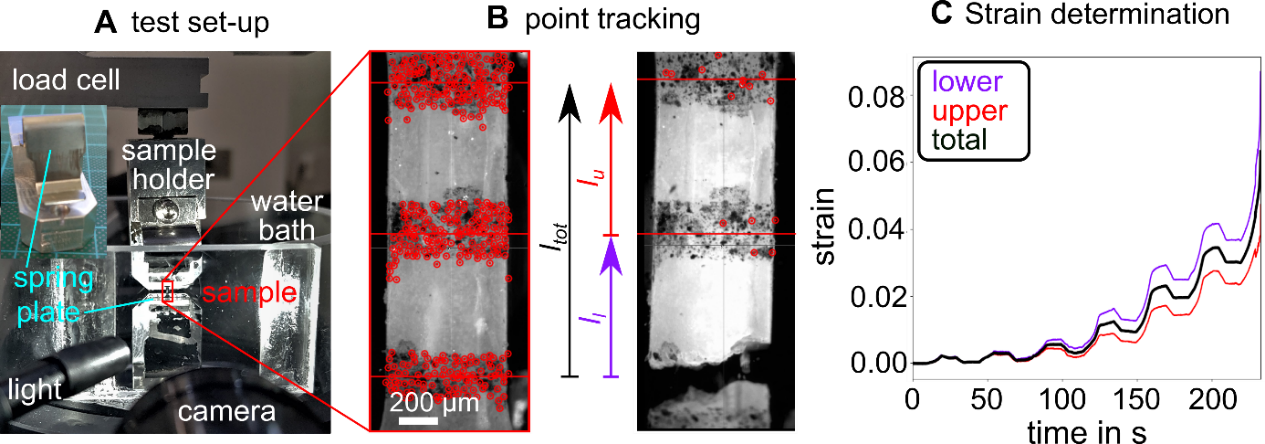


Figure S3: A: test set-up with inset showing tilted sample holder with transistor fitting spring. B: point tracking of total gauge length l_tot_, upper half gauge length l_u_, and lower half gauge length l_l_. C: Determined strain over time, showing load-hold-unload behavior.

## **Measures of specimen geometry**

The average gauge length of cortical bone samples was (1.77 ± 0.13) mm for the control group and (1.78 ± 0.19) mm for the fracture group. Average widths were (0.59 ± 0.08) mm and (0.60 ± 0.07) mm for control and fracture groups, respectively. Specimen thickness was significantly (p = 0.045) smaller for control (0.29 ± 0.08) mm, compared to fracture samples (0.32 ± 0.09) mm. Similarly average area was significantly smaller for control (0.17 ± 0.05) mm^2^ vs. fracture (0.19 ± 0.06) mm^2^ samples. Bone volume to total volume (BVTV) was 0.97 ± 0.02 for control and 0.98 ± 0.02 for fracture samples (n.s.).

## **Representative results trabecular vs. cortical bone tissue**

Selected boxplots of the material and mechanical parameters presented in Table 3 (main manuscript) are illustrated in Figure S4. Hereby, all parameters (long-term and instantaneous modulus, viscosity, yield stress, strain at failure, and damage at end) were significantly different between the two tissues.


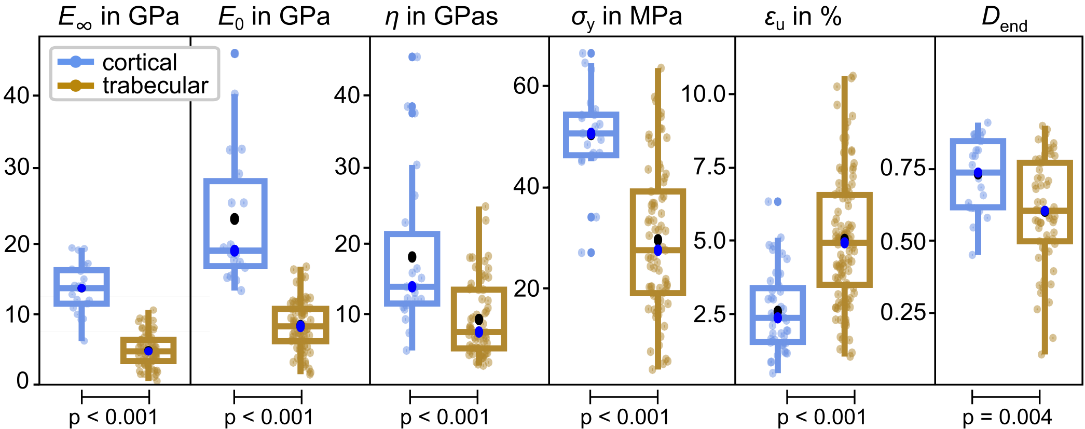


Figure S4: Boxplots for comparison of cortical and trabecular bone tissue, obtained from the same donors. Parameters: long-term modulus $E_{\infty}$, instantaneous modulus $E_{0}$, viscosity $\eta$, yield stress $\sigma_{Y}$, apparent ultimate strain $\hat{\varepsilon}_{u}$, damage parameter at end $D_{end}$. Indicated data points (bright dots), outliers (solid dots) whiskers (minimum, maximum), box (interquartile range), median (blue dot), mean (black dot).

## **Effect of fracture and gender onto material and mechanical properties**

Table S1 demonstrates descriptive statistics (Mann-Whitney-U Test) for grouping based on low-trauma fracture for males and females separately. While most parameters were not differently affected by fracture and gender, yield stress was significantly affected only in males and long-term modulus only in females (with a trend of ultimate strain and mean tissue mineral density).

Table S 1: Descriptive statistics (p values determined with a Mann-Whitney U Test) for grouping based on low-trauma fracture (FRAC) vs. control (CTRL) and gender. Mean values ± std. p: P-values are shown separately for trabecular and cortical bone tissue. Rheological material (top), apparent mechanical (middle), and tissue mineral properties (bottom).

|  | **trabecular** | | | | | | **cortical** | | | | | |
| --- | --- | --- | --- | --- | --- | --- | --- | --- | --- | --- | --- | --- |
| **Parameter** | **male** | | | **female** | | | **male** | | | **female** | | |
|  | **CTRL** | **FRAC** | **p** | **CTRL** | **FRAC** | **p** | **CTRL** | **FRAC** | **p** | **CTRL** | **FRAC** | **p** |
| *E*_∞_ , GPa | 4.9 ± 2.3 | 4.9 ± 2.8 | 0.896 | 4.7 ± 2.3 | 4.5 ± 1.8 | 0.888 | 13.6 ± 4.3 | 12.2 ± 3.9 | 0.346 | 12.7 ± 3.8 | 10.4 ± 3.5 | **0.026** ^a^ |
| *E*_0_ , GPa | 8.4 ± 3.8 | 7.8 ± 3.9 | 0.735 | 7.8 ± 3.3 | 7.9 ± 3.3 | 0.814 | 23.6 ± 8.9 | 26.4 ± 13.8 | 0.815 | 19.0 ± 5.8 | 16.7 ± 5.9 | 0.147 |
| *σ*_y_ , MPa | 29 ± 15 | 36 ± 11 | **0.041**^a^ | 29 ± 15 | 30 ± 13 | 0.555 | 52 ± 9 | 45 ± 8 | 0.115 | 48 ± 10 | 46 ± 10 | 0.346 |
| *p* | 39 ± 26 | 43 ± 33 | 0.822 | 39 ± 32 | 39 ± 29 | 0.988 | 199 ± 125 | 133 ± 94 | 0.185 | 180 ± 108 | 159 ± 105 | 0.591 |
| *σ*_u_ , MPa | 90 ± 32 | 93 ±29 | 0.651 | 88 ± 38 | 100 ± 37 | 0.263 | 67 ± 22 | 61 ± 16 | 0.297 | 62 ± 15 | 61 ± 19 | 0.574 |
| *η*, GPas | 8.5 ± 5.1 | 9.0 ± 4.7 | 0.546 | 8.5 ± 4.6 | 7.7 ± 5.7 | 0.192 | 16.6 ± 10.1 | 14.1 ± 9.4 | 0.387 | 15.0 ± 11.3 | 19.0 ± 10.9 | 0.337 |
| tan*δ* | 0.023 ± 0.014 | 0.020 ± 0.016 | 0.385 | 0.018 ± 0.013 | 0.021 ± 0.013 | 0.333 | 0.072 ± 0.076 | 0.155 ± 0.170 | 0.445 | 0.077 ± 0.116 | 0.037 ± 0.054 | 0.397 |
| *D*_max_ | 0.47 ± 0.25 | 0.45 ± 0.24 | 0.730 | 0.45 ± 0.25 | 0.47 ± 0.25 | 0.797 | 0.57 ± 0.24 | 0.56 ± 0.22 | 0.793 | 0.58 ± 0.20 | 0.56 ± 0.17 | 0.784 |
| *D*_end_ | 0.59 ± 0.19 | 0.60 ± 0.16 | 0.973 | 0.60 ± 0.13 | 0.55 ± 0.21 | 0.588 | 0.74 ± 0.14 | 0.75 ± 0.11 | 1.000 | 0.75 ± 0.10 | 0.71 ± 0.12 | 0.281 |
| $\hat{E}$, GPa | 9.8 ± 5.6 | 7.9 ± 4.8 | 0.124 | 7.1 ± 4.2 | 7.4 ± 4.1 | 0.679 | 18.4 ± 7.2 | 15.9 ± 6.4 | 0.187 | 16.6 ± 5.5 | 14.7 ± 5.4 | 0.075 |
| $\hat{\varepsilon}_{y}$, % | 0.19 ±0.16 | 0.23 ± 0.20 | 0.435 | 0.25 ± 0.16 | 0.30 ± 0.22 | 0.479 | 0.29 ± 0.39 | 0.29 ± 0.21 | 0.226 | 0.23 ± 0.14 | 0.27 ± 0.19 | 0.525 |
| $\hat{\varepsilon}_{u}$, % | 4.7 ± 2.2 | 5.5 ± 2.5 | 0.142 | 5.3 ± 2.2 | 5.5 ± 2.3 | 0.602 | 3.0 ± 1.4 | 2.5 ± 1.3 | 0.191 | 2.3 ± 1.1 | 2.8 ± 1.2 | 0.065 ^b^ |
| $\hat{W}_{\mathrm{el}}$, MJ/m^3^ | 0.015 ± 0.017 | 0.021 ± 0.023 | 0.268 | 0.021 ± 0.018 | 0.024 ± 0.022 | 0.690 | 0.15 ± 0.29 | 0.15 ± 0.16 | 0.503 | 0.10 ± 0.09 | 0.12 ± 0.13 | 0.582 |
| $\hat{W}_{\mathrm{py}}$, MJ/m^3^ | 2.8 ± 1.7 | 3.3 ±2.0 | 0.288 | 3.3 ± 2.1 | 3.5 ± 1.9 | 0.402 | 1.8 ± 1.4 | 1.4 ± 0.9 | 0.531 | 1.3 ± 0.8 | 1.6 ± 0.9 | 0.091 |
| TMD_mean_ , mgHA/cm^3^ | 969 ± 37 | 966 ± 36 | 0.750 | 953 ± 33 | 961 ± 34 | 0.289 | 1042 ± 26 | 1043 ± 17 | 0.611 | 1047 ± 22 | 1033 ± 30 | 0.058 ^b^ |
| TMD_std_ , mgHA/cm^3^ | 173 ± 13 | 173 ± 9 | 0.963 | 176 ± 9 | 175 ± 8 | 0.584 | 155 ± 12 | 153 ± 11 | 0.537 | 150 ± 6 | 149 ± 8 | 0.319 |

^a^ significant p values (< 0.05), marked bold

^b^ p -values indicating a tendency (0.05 < p < 0.07).

## **Effect of age onto material and mechanical properties**

Age had a significant positive effect on ultimate strain
$\hat{\varepsilon}_{u}$for cortical bone tissue (see Table S1). In trabecular bone age had a significant negative effect on damage at maximum stress *D*_max_ and loss tangent.

Table S 2: Effect of age on rheological material (top), apparent mechanical (middle), and tissue mineral properties (bottom) for trabecular and cortical bone tissue. p values determined with a Linear Mixed Effects Model with the effect of low-trauma fracture, age and gender. The present table represents the p-value for the effect of gender. The effect of low-trauma fracture is provided in Table 2, the effect of gender in Table 3.

| **Parameter** | **trabecular**  **p - value** | **cortical**  **p - value** |
| --- | --- | --- |
| *E*_∞_ | 0.599 | 0.445 |
| *E*_0_ | 0.300 | 0.174 |
| *σ*_y_ | 0.094 | 0.469 |
| *p* | 0.256 | ^b^ |
| *σ*_u_ | 0.268 | 0.940 |
| *η* | 0.210 | 0.889 |
| tan*δ* | **< 0.001** ^a^ | 0.235 |
| *D*_max_ | **0.016** ^a^ | 0.302 |
| *D*_end_ | 0.640 | 0.546 |
| $\hat{E}$ | 0.796 | 0.855 |
| $\hat{\varepsilon}_{y}$ | 0.167 | ^b^ |
| $\hat{\varepsilon}_{u}$ | 0.132 | **0.014** ^a^ |
| $\hat{W}_{\mathrm{el}}$ | 0.094 | 0.558 |
| $\hat{W}_{\mathrm{py}}$ | 0.175 | 0.077 |
| TMD_mean_ | 0.706 | 0.892 |
| TMD_std_ | 0.583 | 0.904 |

^a^ significant p values (< 0.05), marked bold.

^b^ the model did not converge for these entries.

## **Correlations between donor BMD with material and mechanical properties**

In classification 2, donor BMD was used as a continuous, independent variable, instead of a binary classification with a low-trauma fracture as grouping variable. Table S2 demonstrates the Pearson correlation coefficient between donor BMD and all rheological material, apparent mechanical, and tissue mineral properties.

Table S3: Pearson correlation coefficient (r) and p value (p) of rheological material, apparent mechanical, and tissue mineral properties with respect to donor BMD.

|  | **cortical** | | **trabecular** | |
| --- | --- | --- | --- | --- |
| **Parameter** | **r** | **p** | **r** | **p** |
| *E*_∞_ | 0.29 | 0.26 | 0.10 | 0.68 |
| *E*_0_ | 0.24 | 0.35 | 0.13 | 0.59 |
| *σ*_y_ | 0.21 | 0.41 | 0.08 | 0.75 |
| *p* | **0.51** ^a^ | 0.05 | -0.05 | 0.83 |
| *σ*_u_ | 0.21 | 0.41 | -0.44 | 0.05 |
| *η* | -0.30 | 0.25 | 0.04 | 0.86 |
| tan*δ* | 0.21 | 0.44 | 0.24 | 0.30 |
| *D*_max_ | 0.33 | 0.37 | 0.38 | 0.11 |
| *D*_end_ | **0.56** ^a^ | 0.02 | 0.25 | 0.31 |
| $\hat{E}$ | 0.36 | 0.10 | 0.09 | 0.70 |
| $\hat{\varepsilon}_{y}$ | 0.04 | 0.88 | -0.11 | 0.64 |
| $\hat{\varepsilon}_{u}$ | 0.11 | 0.65 | -0.26 | 0.27 |
| $\hat{\sigma}_{\max}$ | -0.08 | 0.74 | -0.09 | 0.69 |
| $\hat{W}_{\mathrm{el}}$ | 0.05 | 0.82 | -0.17 | 0.48 |
| $\hat{W}_{\mathrm{py}}$ | 0.04 | 0.87 | -0.19 | 0.43 |
| TMD_mean_ | 0.12 | 0.60 | -0.10 | 0.67 |
| TMD_std_ | 0.06 | 0.81 | 0.03 | 0.90 |
| E_1L_ | 0.32 | 0.16 | 0.10 | 0.67 |
| E_2L_ | 0.38 | 0.09 | 0.02 | 0.94 |
| E_3L_ | -0.19 | 0.42 | -0.07 | 0.78 |
| E_4L_ | 0.13 | 0.57 | -0.08 | 0.73 |
| E_5L_ | 0.03 | 0.90 | -0.02 | 0.92 |
| E_6L_ | -0.01 | 0.96 | -0.12 | 0.62 |
| E_7L_ | **-0.53** ^a^ | 0.11 | 0.23 | 0.38 |
| E_1U_ | -0.08 | 0.74 | 0.25 | 0.30 |
| E_2U_ | -0.45 | 0.05 | 0.44 | 0.05 |
| E_3U_ | -0.27 | 0.29 | 0.10 | 0.68 |
| E_4U_ | 0.09 | 0.74 | 0.06 | 0.81 |
| E_5U_ | -0.08 | 0.77 | -0.12 | 0.64 |
| E_6U_ | **-0.72** ^a^ | 0.03 | 0.20 | 0.42 |
| E_7U_ |  |  | 0.44 | 0.09 |

*^a^ strong correlations (r > 0.5) are indicated bold*

## **Coefficient of variation of material and mechanical properties**

For comparison to previous literature, and to demonstrate the large variation of obtained parameters, Table S3 indicates the coefficient of variation (standard deviation divided by mean) for rheological material and apparent mechanical properties.

Table S4: Coefficient of variation (CV) values of rheological material and apparent mechanical properties

| **Parameter** | **trabecular** | **cortical** |
| --- | --- | --- |
| *E*_∞_ | 0.48 | 0.33 |
| *E*_0_ | 0.44 | 0.43 |
| *σ*_y_ | 0.45 | 0.20 |
| *p* | 0.74 | 0.64 |
| *σ*_u_ | 0.37 | 0.28 |
| *η* | 0.60 | 0.64 |
| tan*δ* | 0.66 | 1.45 |
| *D*_max_ | 0.53 | 0.35 |
| *D*_end_ | 0.31 | 0.16 |
| $\hat{E}$ | 0.59 | 0.37 |
| $\hat{\varepsilon}_{y}$ | 0.78 | 0.86 |
| $\hat{\varepsilon}_{u}$ | 0.44 | 0.47 |
| $\hat{\sigma}_{\max}$ | 0.31 | 0.21 |
| $\hat{W}_{\mathrm{el}}$ | 0.98 | 1.32 |
| $\hat{W}_{\mathrm{py}}$ | 0.59 | 0.66 |

## **Intra donor variation of material and mechanical properties**

Intra donor variation of rheological material and apparent mechanical properties was determined as coefficient of variation (CV: standard deviation divided by mean) for each donor. Table S4 provides the mean of the CV across all donors with respect to low-trauma fracture (separately for cortical and trabecular bone tissue). Only yield stress indicated a significantly lower intra donor variability in donors with a low trauma fracture. In contrast all other variables showed no significant difference (except of a trend of higher TMD variability in donors with a low trauma fracture).

Intra donor variability of mechanical and material properties (*E*_∞_, *E*_0_, *σ*_y_, tan*δ*, *D*_max_, *D*_end_, $\hat{E}$, $\hat{\sigma}_{\max}$, $\hat{W}_{\mathrm{py}}$, TMD_mean_) was significantly lower in cortical bone than in trabecular bone specimens (see Table S5). Hence, cortical bone is a significantly more homogeneous tissue within an individual donor, than trabecular bone.

Table S5: Effect of intra donor variation of rheological material and apparent mechanical properties onto low-trauma fracture. Variation was determined as coefficient of variation (CV) for each donor and mean values across all donors are presented. P-values were determined with a Mann-Whitney U Test, separately for cortical and trabecular bone tissue (CTRL vs. FRAC).

|  | **trabecular** | | | **cortical** | | |
| --- | --- | --- | --- | --- | --- | --- |
| **Parameter** | **CTRL** | **FRAC** | **p** | **CTRL** | **FRAC** | **p** |
| *E*_∞_ | 0.48 | 0.43 | 0.481 | 0.34 | 0.28 | 0.549 |
| *E*_0_ | 0.45 | 0.43 | 0.739 | 0.25 | 0.33 | 0.423 |
| *σ*_y_ | 0.50 | 0.33 | **0.011** ^a^ | 0.20 | 0.15 | 0.897 |
| *p* | 0.79 | 0.66 | 0.579 | 0.56 | 0.56 | 1.000 |
| *σ*_u_ | 0.32 | 0.35 | 0.796 | 0.29 | 0.22 | 0.237 |
| *η*, | 0.57 | 0.58 | 0.971 | 0.54 | 0.51 | 1.000 |
| tan*δ* | 0.48 | 0.52 | 0.863 | 0.82 | 0.78 | 1.000 |
| *D*_max_ | 0.57 | 0.55 | 0.780 | 0.31 | 0.28 | 0.295 |
| *D*_end_ | 0.25 | 0.32 | 0.549 | 0.13 | 0.11 | 0.515 |
| $\hat{E}$ | 0.53 | 0.51 | 0.853 | 0.28 | 0.33 | 0.346 |
| $\hat{\varepsilon}_{y}$ | 0.62 | 0.68 | 0.315 | 0.64 | 0.66 | 0.582 |
| $\hat{\varepsilon}_{u}$ | 0.43 | 0.43 | 1.000 | 0.38 | 0.37 | 0.923 |
| $\hat{\sigma}_{\max}$ | 0.30 | 0.29 | 0.739 | 0.14 | 0.13 | 1.000 |
| $\hat{W}_{\mathrm{el}}$ | 0.82 | 0.89 | 0.888 | 0.89 | 0.90 | 0.674 |
| $\hat{W}_{\mathrm{py}}$ | 0.61 | 0.52 | 0.089 | 0.51 | 0.42 | 0.283 |
| TMD_mean_ | 0.02 | 0.03 | 0.052 ^b^ | 0.01 | 0.02 | 0.497 |
| TMD_std_ | 0.05 | 0.04 | 0.796 | 0.05 | 0.04 | 0.821 |

^a^ significant p values (< 0.05), marked bold

^b^ p -values indicating a tendency (0.05 < p < 0.07)

Table S 6: Effect of intra donor variation of rheological material and apparent mechanical properties with respect to tissue type. Variation was determined as coefficient of variation (CV) for each donor and mean values across all donors are presented. P-values were determined with a Mann-Whitney U Test, for pooled samples (CTRL and FRAC) for each tissue type (trabecular vs. cortical).

|  | **trabecular** | | |
| --- | --- | --- | --- |
| **Parameter** | **trabecular** | **cortical** | **p** |
| *E*_∞_ | 0.45 | 0.31 | **0.022** ^a^ |
| *E*_0_ | 0.44 | 0.29 | **0.002** ^a^ |
| *σ*_y_ | 0.42 | 0.17 | **< 0.001** ^a^ |
| *p* | 0.73 | 0.56 | 0.105 |
| *σ*_u_ | 0.33 | 0.26 | 0.099 |
| *η*, | 0.57 | 0.52 | 0.517 |
| tan*δ* | 0.50 | 0.80 | **0.031** ^a^ |
| *D*_max_ | 0.56 | 0.29 | **< 0.001** ^a^ |
| *D*_end_ | 0.29 | 0.12 | **< 0.001** ^a^ |
| $\hat{E}$ | 0.52 | 0.31 | **< 0.001** ^a^ |
| $\hat{\varepsilon}_{y}$ | 0.64 | 0.65 | 0.980 |
| $\hat{\varepsilon}_{u}$ | 0.43 | 0.38 | 0.257 |
| $\hat{\sigma}_{\max}$ | 0.29 | 0.14 | **< 0.001** ^a^ |
| $\hat{W}_{\mathrm{el}}$ | 0.85 | 0.90 | 0.685 |
| $\hat{W}_{\mathrm{py}}$ | 0.57 | 0.46 | **0.019** ^a^ |
| TMD_mean_ | 0.03 | 0.02 | **< 0.001** ^a^ |
| TMD_std_ | 0.05 | 0.04 | 0.378 |

^a^ significant p values (< 0.05), marked bold

## **Discussion of geometric sample size and anatomical location**

Although a previous study (4) reported a size dependency of obtained mechanical properties for samples below 0.56 mm thickness in three-point bending tests of cortical tibial shaft samples, this effect was not reported to be present in the femoral neck (5). A possible explanation might be the different microstructure in the femoral neck, with a much lower relative osteon area and dimension, compared to the shaft (6). Based on these previous findings, we successfully developed a sample preparation procedure for micro-tensile specimens, as presented in this communication, scaled according to ASTM D1708 – 18. Because of the required dimensions and the 3D curvature of the femoral neck, sample thickness was limited to 0.3 mm, which is approximately two times the size of an osteon diameter (130 to 190 µm (6)). The inferomedial region was chosen instead of the thinner superolateral neck to obtain more samples per donor. Tensile testing was preferred over three-point bending because of a homogenous stress state and a higher sensitivity to flaws. Further, the current study used the same tensile test protocol that was previously established for individual trabeculae obtained from the femoral head and neck (3). This enabled a direct comparison between trabecular and cortical bone tissue. Moreover, the scale of individual trabeculae and corresponding cortical bone samples was similar. While cortical bone samples were physically larger, they consisted of the same corresponding morphological units (several osteons (7)), as single trabeculae (several trabecular packets (8)). Knowledge of the mechanical properties at this hierarchical level is important, since they are required as input parameters for computer simulations, such as finite element analysis (FEA).

## **Discussion of effect of age and tissue mineralization**

A further cause of previously reported differences of mechanical properties of donors with osteoporosis (9) might be that young control (on average 34.8 years old) donors were compared with elderly osteoporotic (on average 80.2 years old) ones. Milovanovic et al. (10) demonstrated that bone mineral density distribution, mineral to matrix ratio, and reference point indentation (RPI) was significantly different between young and osteoporotic donors, but not between aged and osteoporotic donors. Jenkins et al. also found significant differences in RPI measures between osteoporotic and control donors (partially the same donors as in the current study) in the circumferential femoral neck, whereby osteoporotic donors were significantly older than controls. Similarly, Bernard et al. (11) demonstrated that osteon morphology changes in ageing and osteoporosis, compared to young donors, but is not different between aged and osteoporotic donors. In accordance, Morgan et al. (12) stated in a recent review that the effects of aging are not readily distinguishable from those of osteoporosis. In the present study, a larger donor age was associated with a decrease of damage accumulation and loss tangent in trabecular bone, whereas ultimate strain was significantly larger in cortical bone for older donors (see Table S2). In contrast, all other investigated parameters were not affected by age, meaning that there is a small, but significant effect. Hereby it has to be mentioned that the age range studied was small (57 – 97 years) and only included post-menopausal donors in the female cohort.

In the present study, µCT was used to assesses mean tissue mineralization, whereas quantitative back scattered electron imaging (qBEI) is more commonly used. However, previously Mashiatulla et al. (13) demonstrated that mean tissue mineralization obtained from both measurements correlated strongly. In the present study, mean tissue mineral density was not different between osteoporotic and control donors, in accordance with qBEI data on trans iliac core biopsies (14) and the cortex of the proximal femur (10). In more detail, Milovanovic et al. (10) found no difference in tissue mineralization between osteoporotic and aged donors, but in comparison to young donors. In contrast, Fratzl-Zelman et al. determined a significant decrease in mineralization density in the femoral neck of age-matched osteoporotic donors (15). A possible explanation for this discrepancy could be that donor age was assessed, but not tissue age, which is likely more determining material properties (16). Aging and osteoporosis causes an increase of cortical porosity (17,18), a decrease of mineralization density (10,19,20), an accumulation of microdamage (21,22), an increased concentration of advanced glycation end products (AGEs) (23,24), and a decrease of tissue strain in the mineralized collagen fibril (9,24). This can be further related to a more brittle apparent mechanical behavior under both conditions (17,24–27) but does not contribute a lot in discriminating the increased risk of fracture in osteoporotic patients, compared to age-matched controls.

**References**

1. Jenkins T. Bioengineering Sciences Research Group A Biomechanical Approach to Improved Fracture Risk Assessment with a Focus on Reference Point Microindentation. University of Southampton; 2015.

2. Jenkins T, Coutts L V., D’Angelo S, Dunlop DG, Oreffo ROCC, Cooper C, et al. Site-Dependent Reference Point Microindentation Complements Clinical Measures for Improved Fracture Risk Assessment at the Human Femoral Neck. J Bone Miner Res. 2016;31(1):196–203.

3. Frank M, Reisinger AG, Pahr DH, Thurner PJ. Effects of Osteoporosis on Bone Morphometry and Material Properties of Individual Human Trabeculae in the Femoral Head. JBMR Plus. 2021;5(6):1–14.

4. Choi K, Kuhn JL, Ciarelli MJ, Goldstein SA. The elastic moduli of human subchondral, trabecular, and cortical bone tissue and the size-dependency of cortical bone modulus. J Biomech. 1990;23(11):1103–13.

5. Gastaldi D, Baleani M, Fognani R, Airaghi F, Bonanni L, Vena P. An experimental procedure to perform mechanical characterization of small-sized bone specimens from thin femoral cortical wall. J Mech Behav Biomed Mater [Internet]. 2020;112(August):104046. Available from: https://doi.org/10.1016/j.jmbbm.2020.104046

6. Tong X, Burton IS, Jurvelin JS, Isaksson H, Kröger H. Iliac crest histomorphometry and skeletal heterogeneity in men. Bone Reports [Internet]. 2017;6:9–16. Available from: http://dx.doi.org/10.1016/j.bonr.2016.11.004

7. Clarke B. Normal bone anatomy and physiology. Clin J Am Soc Nephrol. 2008;3 Suppl 3:131–9.

8. Fratzl P, Gupta HS, Paschalis EP, Roschger P. Structure and mechanical quality of the collagen-mineral nano-composite in bone. J Mater Chem. 2004;14(14):2115–23.

9. Zimmermann EA, Schaible E, Gludovatz B, Schmidt FN, Riedel C, Krause M, et al. Intrinsic mechanical behavior of femoral cortical bone in young, osteoporotic and bisphosphonate-treated individuals in low-and high energy fracture conditions. Sci Rep. 2016;6(February):1–12.

10. Milovanovic P, Zimmermann EA, Riedel C, Scheidt A vom, Herzog L, Krause M, et al. Multi-level characterization of human femoral cortices and their underlying osteocyte network reveal trends in quality of young, aged, osteoporotic and antiresorptive-treated bone. Biomaterials. 2015;45:46–55.

11. Bernhard A, Milovanovic P, Zimmermann EA, Hahn M, Djonic D, Krause M, et al. Micro-morphological properties of osteons reveal changes in cortical bone stability during aging, osteoporosis, and bisphosphonate treatment in women. Osteoporos Int. 2013;24(10):2671–80.

12. Morgan EF, Unnikrisnan GU, Hussein AI. Bone Mechanical Properties in Healthy and Diseased States. Annu Rev Biomed Eng. 2018;20:119–43.

13. Mashiatullaa M, Rossa RD, Sumner DR. Validation of Cortical Bone Mineral Density Distribution using micro-Computed Tomography. Bone. 2017;6(99):53–61.

14. Tjhia CK, Odvina C V., Rao DS, Stover SM, Wang X, Fyhrie DP. Mechanical property and tissue mineral density differences among severely suppressed bone turnover (SSBT) patients, osteoporotic patients, and normal subjects. Bone [Internet]. 2011;49(6):1279–89. Available from: http://dx.doi.org/10.1016/j.bone.2011.09.042

15. Fratzl-Zelman N, Roschger P, Gourrier A, Weber M, Misof BM, Loveridge N, et al. Combination of Nanoindentation and Quantitative Backscattered Electron Imaging Revealed Altered Bone Material Properties Associated with Femoral Neck Fragility. Calcif Tissue Int. 2009;85:335–43.

16. Nyman JS, Granke M, Singleton RC, Pharr GM. Tissue-Level Mechanical Properties of Bone Contributing to Fracture Risk. Curr Osteoporos Rep [Internet]. 2016;14(4):138–50. Available from: http://dx.doi.org/10.1007/s11914-016-0314-3

17. Malo MKH, Rohrbach D, Isaksson H, Töyräs J, Jurvelin JS, Tamminen IS, et al. Longitudinal elastic properties and porosity of cortical bone tissue vary with age in human proximal femur. Bone [Internet]. 2013;53(2):451–8. Available from: http://dx.doi.org/10.1016/j.bone.2013.01.015

18. Mirzaali MJ, Schwiedrzik JJ, Thaiwichai S, Best JP, Michler J, Zysset PK, et al. Mechanical properties of cortical bone and their relationships with age, gender, composition and microindentation properties in the elderly. Bone [Internet]. 2016;93:196–211. Available from: http://dx.doi.org/10.1016/j.bone.2015.11.018

19. Brennan MA, Gleeson JP, O’Brien FJ, McNamara LM. Effects of ageing, prolonged estrogen deficiency and zoledronate on bone tissue mineral distribution. J Mech Behav Biomed Mater [Internet]. 2014;29:161–70. Available from: http://dx.doi.org/10.1016/j.jmbbm.2013.08.029

20. Roschger P, Paschalis EP, Fratzl P, Klaushofer K. Bone mineralization density distribution in health and disease. Bone. 2008;42(3):456–66.

21. Schaffler MB, Choi K, Milgrom C. Aging and matrix microdamage accumulation in human compact bone. Bone. 1995;17(6):521–5.

22. Burr DB, Forwood MR, Fyhrie DP, Martin RB, Schaffler MB, Turner CH. Bone Microdamage and Skeletal Fragility in Osteoporotic and Stress Fractures. J Bone Miner Res [Internet]. 1997;12(1):6–15. Available from: http://dx.doi.org/10.1016/S0278-5919(05)70020-X

23. Wang X, Shen X, Li X, Agrawal CM. Age-related Changes in the Collagen Network and Toughness of Bone. Bone. 2002;31(1):1–7.

24. Zimmermann EA, Schaible E, Bale H, Barth HD, Tang SY, Reichert P, et al. Age-related changes in the plasticity and toughness of human cortical bone at multiple length scales. Proc Natl Acad Sci U S A. 2011;108(35):14416–21.

25. Augat P, Schorlemmer S. The role of cortical bone and its microstructure in bone strength. Age Ageing. 2006;35(SUPPL.2):27–31.

26. Lefèvre E, Farlay D, Bala Y, Subtil F, Wolfram U, Rizzo S, et al. Compositional and mechanical properties of growing cortical bone tissue: a study of the human fibula. Sci Rep. 2019;9(1):1–16.

27. Nyman JS, Roy A, Reyes MJ, Wang X. Mechanical behavior of human cortical bone in cycles of advancing tensile strain for two age groups. J Biomed Mater Res - Part A. 2009;89(2):521–9.
